# IOX1 activity as sepsis therapy and an antibiotic against multidrug-resistant bacteria

**DOI:** 10.1038/s41598-021-82377-z

**Published:** 2021-02-03

**Authors:** Su Jin Lee, Jueng Soo You, Amal Gharbi, Yong Joo Kim, Mi Suk Lee, Dong Hwan Kim, Keun Woo Lee, In Duk Jung, Yeong Min Park

**Affiliations:** 1 Department of Immunology, Laboratory of Dendritic Cell Differentiation and Regulation, School of Medicine, Konkuk University, Chungju, Seoul, 380-701 Republic of Korea; 2 Department of Biochemistry, School of Medicine, Konkuk University, Chungju, Seoul, 380-701 Republic of Korea; 3 Dandi Bioscience Inc, 6Th Floor of Real Company, 66, Acha San-ro, Seongdong-gu, Seoul, Republic of Korea; 4 Division of Life Science, Research Institute of Natural Science (RINS), Gyeongsang National University (GNU), 501 Jinju-daero, Jinju, 52828 Republic of Korea; 5 Division of Applied Life Science (BK21 Plus), Research Institute of Natural Science (RINS), Gyeongsang National University (GNU), 501 Jinju-daero, Jinju, 52828 Republic of Korea

**Keywords:** Immunology, Microbiology

## Abstract

Sepsis is caused by organ dysfunction initiated by an unrestrained host immune response to infection. The emergence of antibiotic-resistant bacteria has rapidly increased in the last decades and has stimulated a firm research platform to combat infections caused by antibiotic-resistant bacteria that cannot be eradicated with conventional antibiotics. Strategies like epigenetic regulators such as lysine demethylase (Kdm) has received attention as a new target. Thus, we sought to investigate the epigenetic mechanisms in sepsis pathophysiology with the aim of discovering new concepts for treatment. A transcriptome analysis of dendritic cells during their inflammatory state identified Kdm as a critical molecule in sepsis regulation. Next, 8-hydroxyquinoline-5-carboxylic acid (IOX1) ability to control endotoxemia induced by Lipopolysaccharide and bacterial sepsis was demonstrated. IOX1 has been shown to regulate endotoxemia and sepsis caused by *Escherichia coli* and carbapenem-resistant *Acinetobacter baumannii* and has also contributed to the suppression of multidrug-resistant bacterial growth through the inhibition of DNA Gyrase. These findings show that IOX1 could be a component agent against bacterial sepsis by functioning as a broad-spectrum antibiotic with dual effects.

## Introduction

Despite advancement in medical care, sepsis remains the leading cause of death from infection due to the emergence of antibiotic resistance and the absence of novel treatments for sepsis^1^. A pioneering review highlighted that the mechanisms of epigenetic regulation may be the major lead to immune suppression by averting the proliferation and function of effector immune cells during sepsis progression^2,3^. Histone lysine methylation is a reversible epigenetic modification dependent on the activities of histone lysine methyltransferases and histone lysine demethylases (Kdms)^4,5^. Among them, Kdm4A (JMJD2A) and Kdm6B (JMJD3) have been reported to play an important role in modifying the expression of the central genes of the inflammatory signaling pathway^6,7^. However, with regard to Kdm regulation in sepsis induced by bacteria or endotoxins, basic molecular mechanisms have not been reported. Equally important, most bacteria have two unique type II topoisomerases (topos), DNA gyrase and topoisomerase IV, which catalyze the essential processes for DNA replication and cell survival^8–11^. Of these, DNA gyrase has been an important antimicrobial drug target^12,13^. Thus, quinolones and fluoroquinolones interfering with gyrase functions^14–16^ have attracted recognition as antibacterial agents displaying minimal side effects^17^. It has been reported that 8-hydroxyquinoline-5-carboxylic acid (IOX1) does not require a prodrug form during application and has a broad spectrum of cell permeability, suggesting that it could be an effective Kdm inhibitor^18,19^. Thus, suggests that it might be used as a therapeutic intervention for diseases such as anemia, inflammation and cancer. Therefore, we tested IOX1 in the treatment of sepsis, and we propose it as a new promising broad-spectrum therapeutic agent for sepsis.

## Results

### IOX1 inhibits the immune response via DC maturation induced by LPS

**Figure 1:**
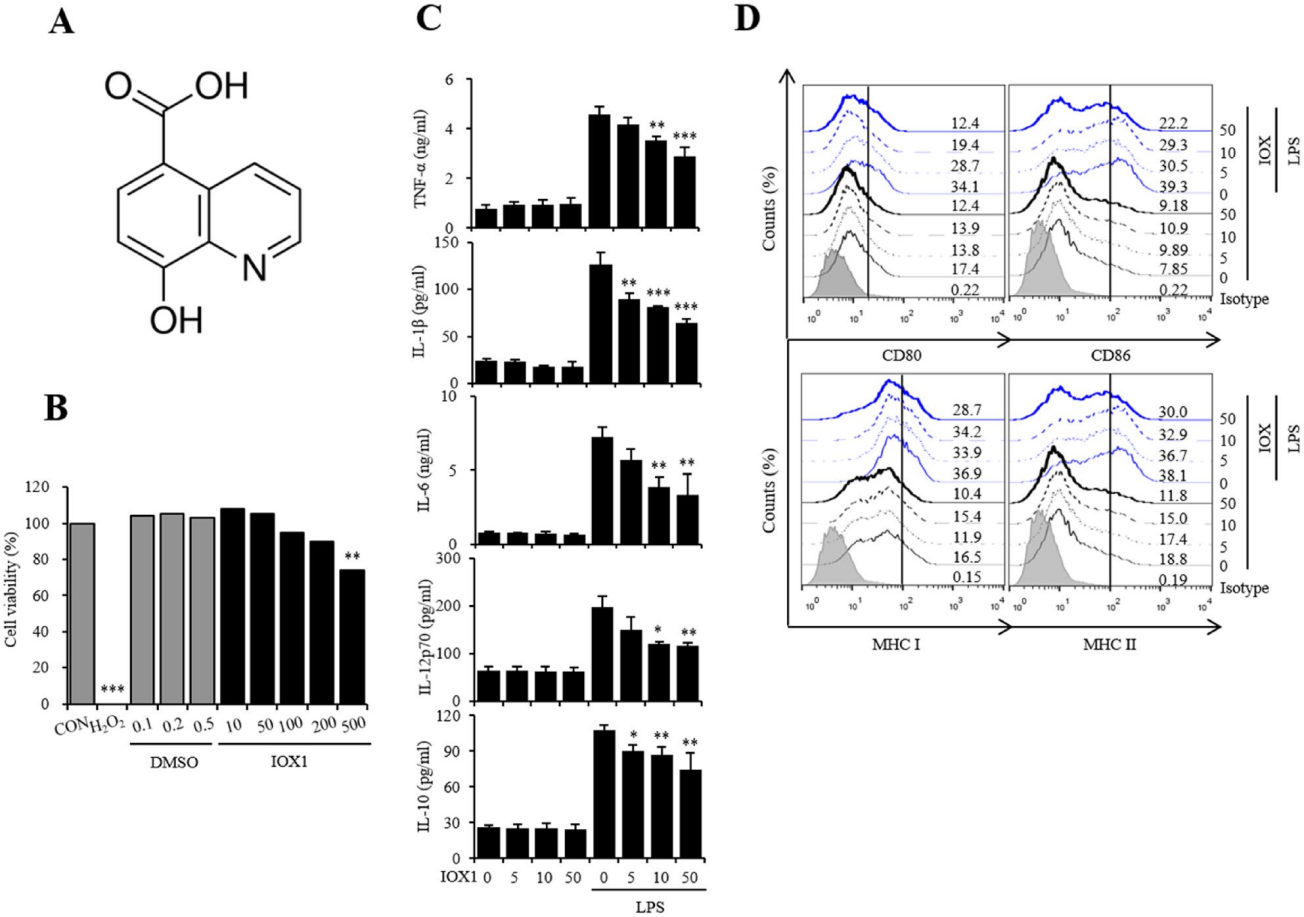
IOX1 suppresses the inflammatory response in LPS-induced DC maturation. (**A**) Chemical structure of IOX1. (**B**) Mouse bone marrow-derived DCs were treated with the indicated concentrations of IOX1, DMSO or H_2_O_2_ (negative control) overnight. The cytotoxicity of IOX1 in DCs was analyzed by a Luminescent Cell Viability Kit. (**C**) DCs treated with IOX1 (50 μM) before or after LPS stimulation (50 ng/ml) at the indicated times. Culture medium was collected, and the TNF-α, IL-1β, IL-6, IL-12p70 and IL-10 levels in the medium were determined by ELISA. (**D**) BMDCs were pretreated for 30 min with the indicated concentrations of IOX1 before stimulation with LPS (50 ng/ml) overnight. The surface molecule expression of BMDCs was analyzed by flow cytometry. The results of one representative experiment out of three experiments are shown. Data are presented as the means ± SEMs. *P < 0.05; **P < *0.01*; and ***P < 0.001 compared to DCs treated with LPS. *n.s.* no significance.

To investigate whether histone lysine methylation is a targetable therapeutic point, we checked the expression change of enzymes that are responsible for histone methylation and found that Kdm4a and Kdm6b are the most upregulated enzymes upon LPS treatment in dendritic cells (DCs) (Fig. S1). Next, we searched for specific inhibitors of Kdm4a and Kdm6b. Given the advantages of IOX1, we selected IOX1 from among a variety of Kdm inhibitors. The structure of IOX1 (8-hydroxyquinoline-5-carboxylic acid) is shown in Fig. 1A. The cytotoxicity of IOX1 was measured after treatment of dendritic cells with the indicated concentration of IOX1 in the same amount of DMSO and a positive control, H_2_O_2_. In the case of DMSO, up to 0.5% of the volume of DMSO used to dissolve IOX1 was found to be noncytotoxic, and in the case of IOX1, no cytotoxicity was observed below 200 μM (Fig. 1B). Therefore, the concentration of IOX1 for DC function analysis in the future should be less than 200 μM. To analyze the effect of IOX1 on LPS-induced DC activation, we investigated the changes in inflammatory cytokine mRNA expression and secretion (Fig. S2 and Fig. 1C) and the expression of surface molecules as maturation markers (Fig. 1D). IOX1 significantly reduced the secretion of TNF-α, IL-1β, IL-6, IL-12p70 and IL-10 induced by LPS (Fig. 1C). The expression of surface molecules, such as CD80, CD86, MHC-I and MHC-II, induced by LPS was also significantly reduced (Fig. 1D). These results indicate that IOX1 can effectively regulate the immune activity of bone marrow DCs induced by LPS.

### IOX1 suppresses the immune response by inhibiting Kdm4a

**Figure 2:**
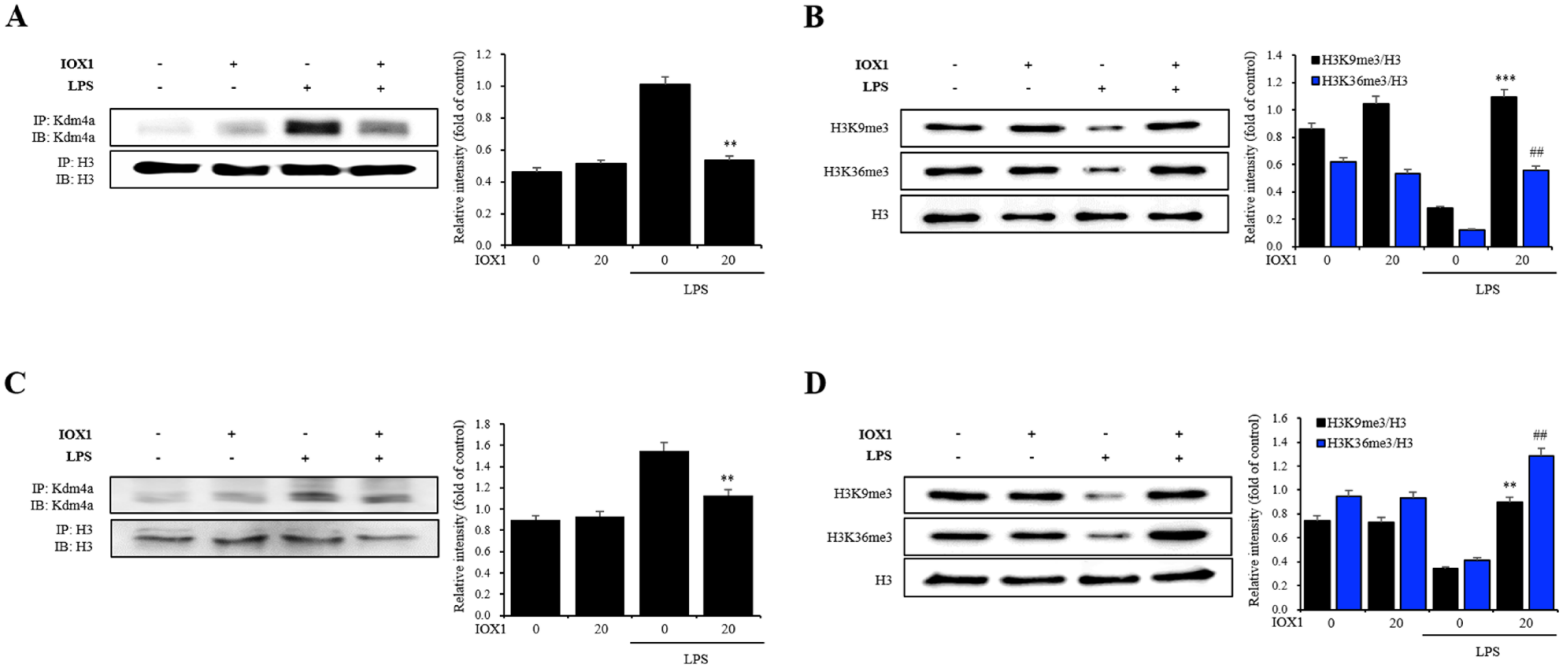
IOX1 reverses the downregulation of histone methylation via Kdm4a modification by LPS in vitro and in vivo. DCs from C57BL/6 mice were stimulated with 50 ng/ml LPS in the presence or absence of 50 μM IOX1 overnight. Lungs from BALB/c mice were injected with 10 mg/kg LPS in the presence or absence of 20 mg/kg IOX1 overnight. Total protein extracts from DCs and lungs treated with IOX1 were used to detect Kdm4a (**A**,**C**), H3K9me3 and H3K36me3 (**B**,**D**) by Western blotting, Histone H3 was used as a loading control (full-length blots are presented in Figs. S10–S13); Immunoprecipitation (IP) assays were performed on DCs treated in the presence or absence of IOX1, using specific antibodies to Kdm4a and histone H3 as controls (full-length blots are presented in Figs. S10 and S12).

To determine whether the effect of IOX1 is caused by inhibition of Kdm, we performed immunoprecipitation with histone H3 and Kdm4a, followed by Western blot. In correlation with the transcriptome data, Kdm4a was highly upregulated upon LPS addition in BMDCs, and the target modifications H3K36me3 and H3K9me3 levels decreased as Kdm4a levels increased (Fig. 2A,B). IOX1 significantly suppressed Kdm4a upregulation and restored H3K36me3 and H3K9me3 levels (Fig. 2A,B). Changes in Kdm4a levels and target histone methylation induced by LPS and IOX1 were mimicked in the lungs of mice (Fig. 2C,D). These results suggest that the inhibitory effect of IOX1 on the immune response occurs via a mechanism of Kdm inhibition.

### IOX1 anti-inflammatory effect on an LPS-induced endotoxicity mouse model

**Figure 3:**
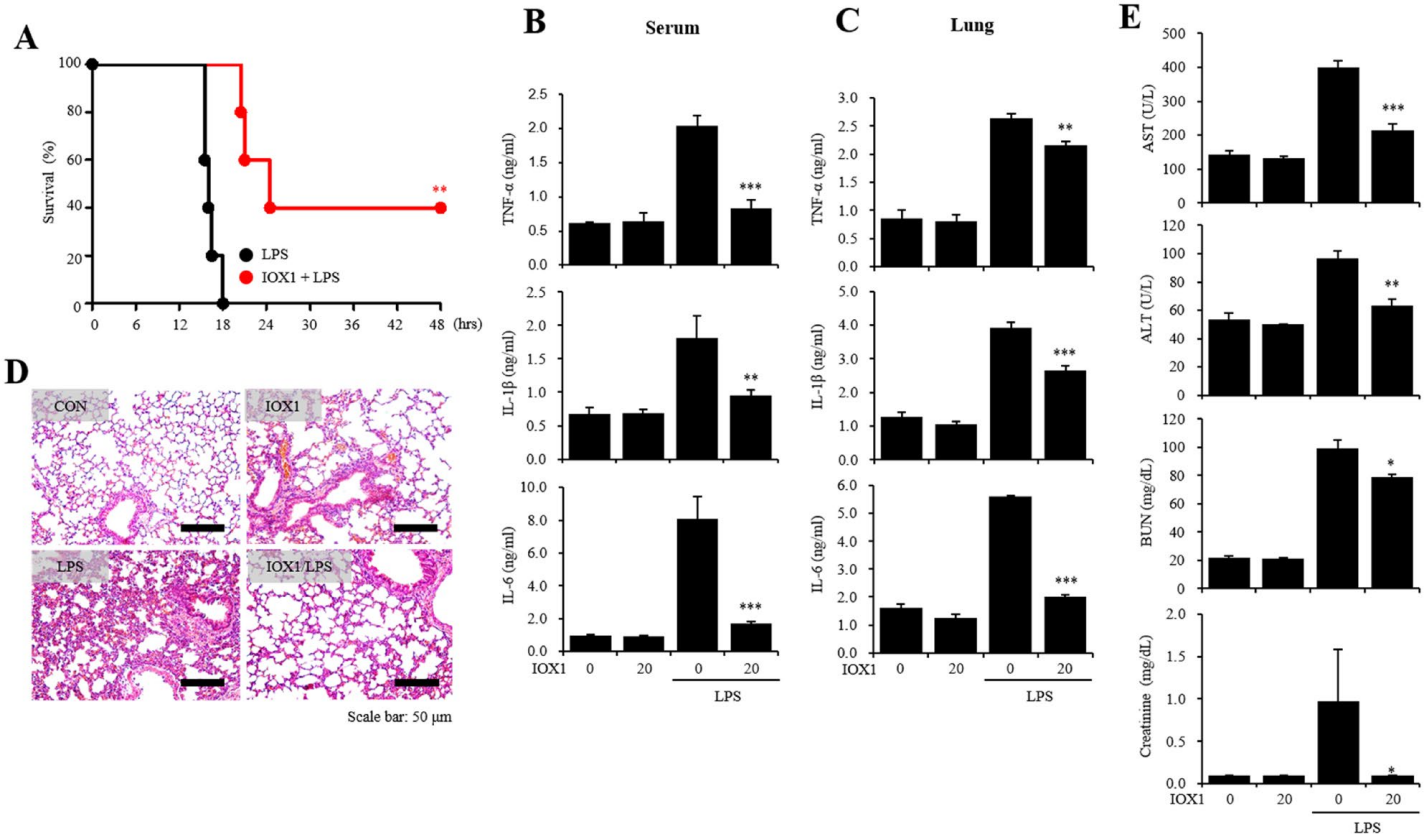
IOX1 anti-inflammatory effects on an LPS-induced endotoxemia mouse model. Six-week-old female BALB/c mice were intraperitoneally injected with IOX1 (20 mg/kg). After 30 min, the mice were i.p. injected with LPS. (**A**) The survival rates of IOX1- and LPS (20 mg/kg)-injected mice were monitored for 48 h. (**B**) The serum of IOX1- and LPS (20 mg/kg)-injected mice were harvested 2 h after LPS injection. The levels of serum proinflammatory cytokines (TNF-α, IL-1β and IL-6) were measured by sandwich ELISA kits. (**C–E**) After overnight incubation, IOX1- and LPS (10 mg/kg)-injected mice were sacrificed for experiments. (**C**) The mouse lungs were homogenized by stainless steel beads. The lung levels of proinflammatory cytokines (TNF-α, IL-1β and IL-6) were measured by sandwich ELISA kits. (**D**) The PMN infiltrations in the lung were stained using the hematoxylin and eosin standard staining method. (**E**) The levels of serum AST, ALT, BUN and creatinine were measured by a laboratory medicine system.

We initiated an in vivo study by validating the anti-inflammatory effect of IOX1. A 30-min pretreatment with IOX1 (20 mg/kg, i.p.) prior to LPS (20 mg/kg, i.p.) injection ameliorated the survival of endotoxemia-induced mice. The survival rate increased within 18 h from 0% in mice injected solely with LPS to 40% in mice pretreated with IOX1 (Fig. 3A). In mouse serum, the increased levels of TNF-α, IL-1β and IL-6 induced by LPS were significantly reduced by IOX1 administration (Fig. 3B). In lung tissues, the expression of TNF-α, IL-1β and IL-6 was increased by LPS injection, and infiltration of PMNs (polymorphonuclear leukocytes) was also significantly reduced by IOX1 administration (Fig. 3C,D). Furthermore, the levels of AST, ALT, BUN and creatinine, biomarkers of organ failure, that were enhanced by LPS injection were reduced by IOX1 administration (Fig. 3E). These results confirmed that IOX1 has an anti-inflammatory effect on the LPS-induced endotoxicity mouse model.

### IOX1 antiseptic effect on an *A. baumannii*-inoculated septic mouse model

**Figure 4:**
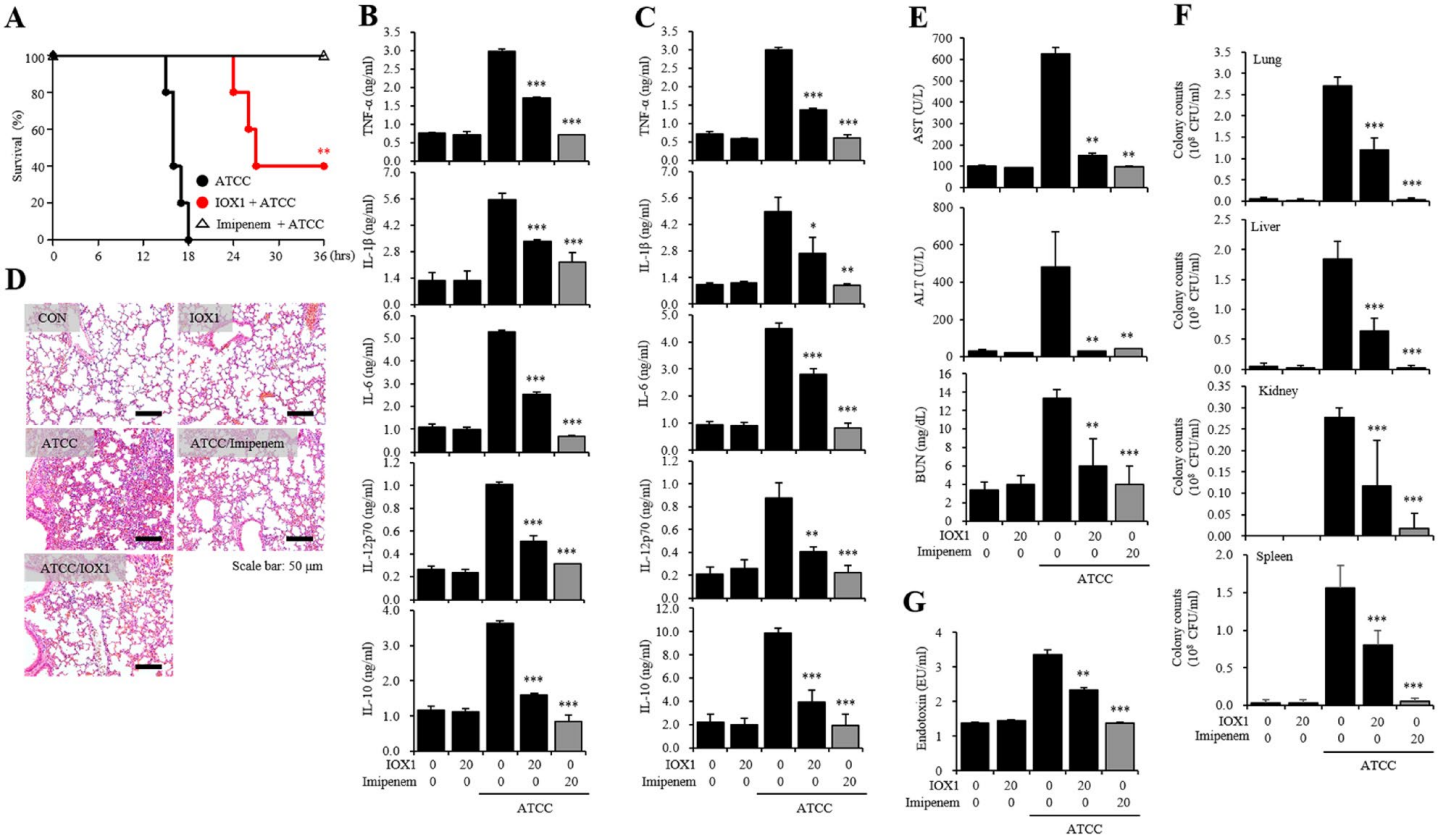
IOX1 antiseptic effect on an *A. baumannii*-inoculated septic mouse model. Six-week-old female BALB/c mice were intraperitoneally injected with IOX1 (20 mg/kg). After 30 min, the mice were i.p. injected with *A. baumannii* (KUMC ATCC 19606, 1.9 × 10^4^ CFU/mouse). (**A**) The survival rates of IOX1- and *A. baumannii*-injected mice were monitored for 48 h. (**B**) The serum of mice was harvested 2 h after *A. baumannii* (9.5 × 10^3^ CFU/mouse) injection in the presence or absence of IOX1. The levels of serum inflammatory cytokines (TNF-α, IL-1β, IL-6, IL-12p70 and IL-10) were measured by sandwich ELISA kits. (**C–G**) After overnight incubation, IOX1- and *A. baumannii* (9.5 × 10^3^ CFU/mouse)-injected mice were sacrificed for experiments. (**C**) The mouse lungs were homogenized by stainless steel beads. The levels of lung inflammatory cytokines (TNF-α, IL-1β, IL-6, IL-12p70 and IL-10) were measured by sandwich ELISA kits. (**D**) PMN infiltrations in the lung were stained using the H&E standard staining method. (**E**) The serum levels of AST, ALT, BUN and creatinine were measured by a laboratory medicine system. (**F**) The mouse lungs, livers, kidneys and spleens were homogenized by stainless steel beads. The lysates were diluted with PBS and incubated on LB agar plates overnight. (**G**) The serum levels of endotoxin were determined by the LAL method and measured at 405 nm.

Furthermore, an enhanced survival rate was observed in an *A. baumannii*-inoculated septic mouse model. Using the carbapenem antibiotic imipenem (1 mg/kg) as a control drug (100% survival rate), intraperitoneal administration of a clinical standard strain of *A. baumannii* (1.9 × 10^4^ CFU/mouse) 30 min after IOX1 administration resulted in reduced sepsis incidence within 18 h, with a 40% increase in the survival rate in the group pretreated with IOX1 (20 mg/kg) compared with that in the group injected only with *A. baumannii* (Fig. 4A). The levels of the proinflammatory cytokines TNF-α, IL-1β, IL-6, IL-12p70, and IL-10 in the serum (Fig. 4B) and in the lungs (Fig. 4C) of the *A. baumannii*-derived sepsis mouse model treated with IOX1 were significantly reduced. H&E staining demonstrated that IOX1 could repress PMN (polymorphonuclear leukocyte) infiltration in the tissue of the corresponding sepsis mouse model (Fig. 4D) and could also mitigate AST, ALT, BUN and creatinine levels in the lungs of the same mice (Fig. 4E). Interestingly, IOX1 showed an effective inhibition of colony growth (CFU) of *A. baumannii* in the major organs. A total repression of colony numbers was observed in the lung, kidney and spleen, and a significant reduction was reported in the liver (Fig. 4F). Additionally, the endotoxin levels in mouse serum were also significantly reduced (Fig. 4G). Notably, IOX1 effects were reproducible in an *E. coli* K1 sepsis mouse model, with a survival rate of 60% (Fig. S3). These results show that IOX1 can act as an endotoxin removal agent and an effective inhibitory molecule against bacterial colony growth, resulting in effective treatment of *A. baumannii*-induced sepsis.

### IOX1 antibiotic effect on gram-negative bacteria

**Figure 5:**
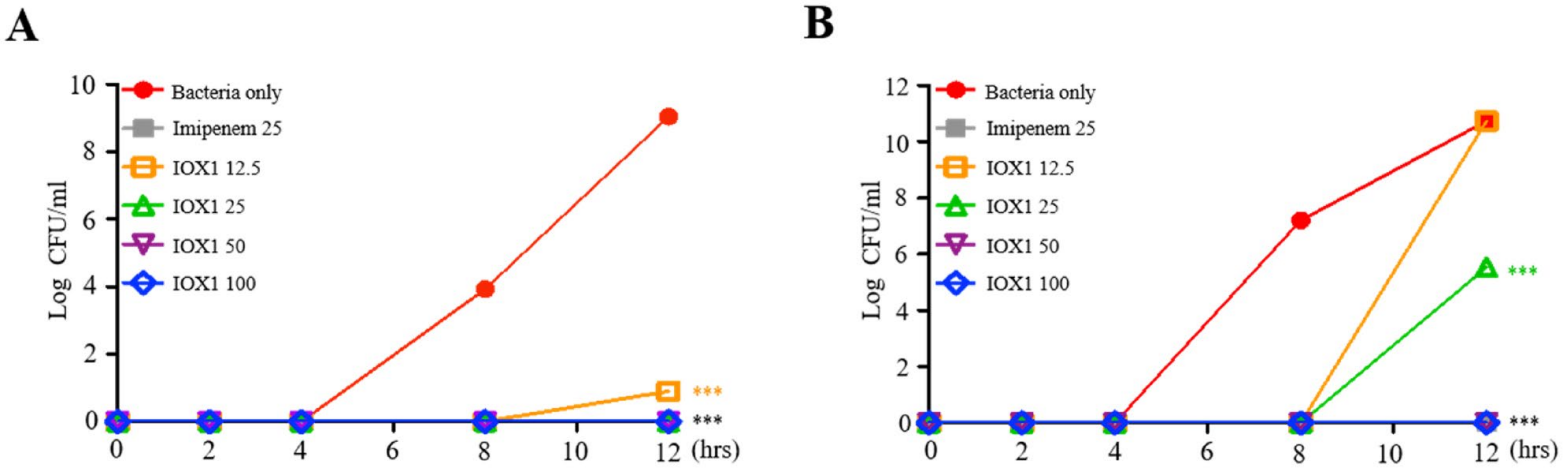
IOX1 antibiotic effect on gram-negative bacteria. (**A**) Bactericidal kinetics of IOX1 against susceptible *DH5a* (4.03 × 10^4^ CFU/ml) and (**B**) *A. baumannii* KUMC.2015.sus (4.3 × 10^4^ CFU/ml).

Next, we sought to elaborate on the data obtained above (Fig. 4F) by investigating the potential antibacterial activity exerted by IOX1 against bacteria. First, we analyzed the time-kill kinetics profile of IOX1 against *E. coli* (*DH5α*) and *A. baumannii* following different addition of concentrations of 12.5, 25, 50 and 100 μg/ml at various checkpoint times of 0, 4, 6, 8, 10 and 12 h. IOX1 completely inhibited the growth of *E. coli* (*DH5α*) at up to 12 h at a concentration of 25 μg/ml, the same concentration for which antibiotic activity was shown for the positive control imipenem (Fig. 5A), and inhibited *A. baumannii* at a concentration of 50 μg/ml (Fig. 5B). IOX1 exhibited inhibitory growth effects on different types of gram-negative bacterium, *S.enteritidis* (Fig. S4A),* S.typhimurium* (Fig. S4B), *K.pneumoniae* (Fig. S4C)*,* and *P.aeruginosa* (Fig. S4D)*,* with complete bacterial growth inhibition at a concentration of 50 μg/ml at up to 12 h. The antibacterial effect of IOX1 was also confirmed for the gram-positive bacterium *S. aureus* (Fig. S5A) and colistin-resistant *AB* (Fig. S5B) in a concentration-dependent manner, with complete bacterial growth inhibition at a concentration of 50 μg/ml at up to 12 h and 8 h, respectively. The antibacterial activity against not only gram-negative bacteria but also gram-positive bacteria was shown. This antibacterial effect against different types of bacteria was further proven in a cecal content injection (CCI) mouse model (Fig. S6). These results indicate that IOX1 exhibits a bactericidal effect that led to a significant reduction in the bacterial colony count and endotoxin levels.

### IOX1 inhibits DNA gyrase activity similarly to quinolone antibiotics

**Figure 6:**
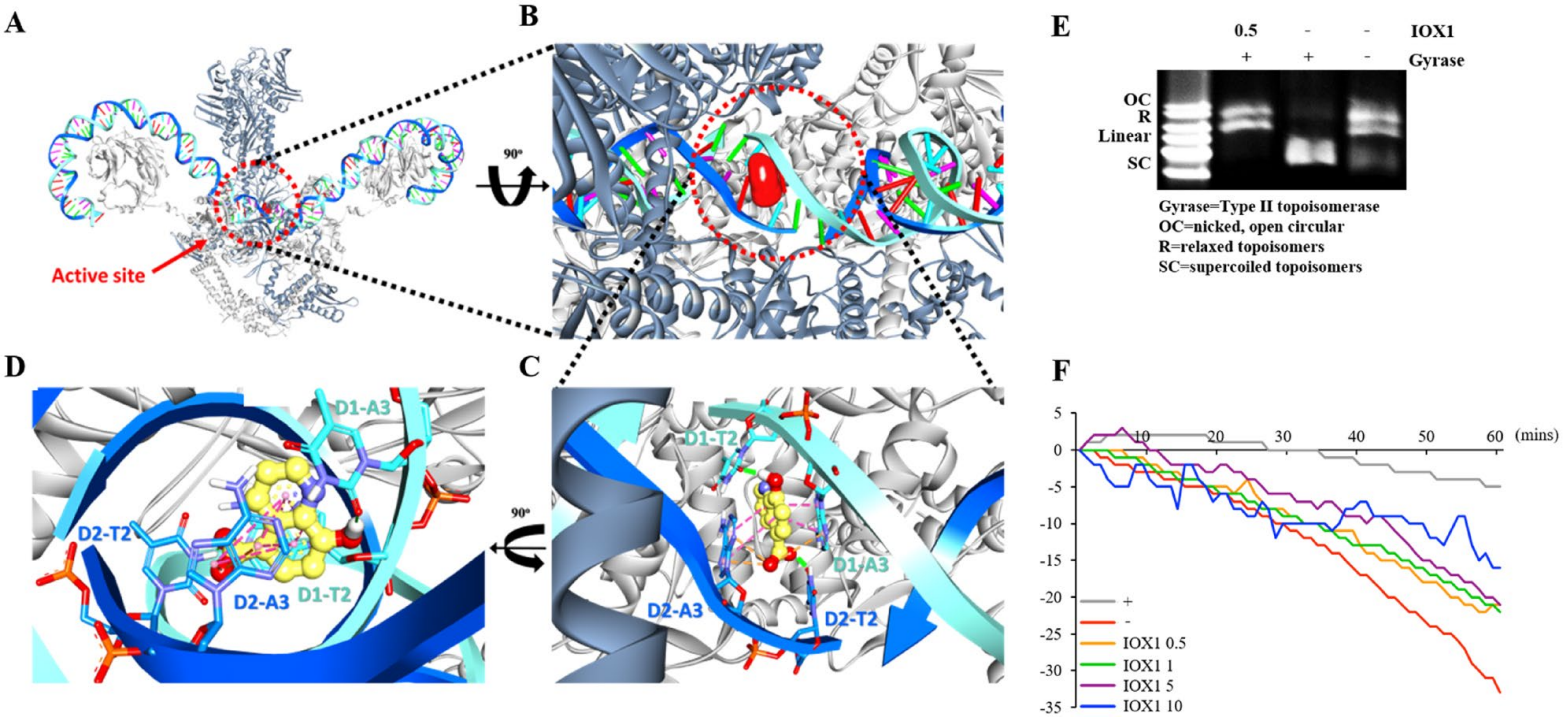
Mode of action of IOX1 as an antibiotic against bacteria. (**A**) Front views of the 3D structure of *E. coli* DNA gyrase. DNA chains 1 and 2 are shown in cyan and blue, and subunits A and B are shown in gray and dark gray, respectively. The active site is shown in the red cycle, and the IOX1 surface is shown in red. (**B**) Close-up view of the active site of *E. coli* DNA gyrase after 90° rotation for a better view. (**C**) Close-up view of the active site in *E. coli* DNA gyrase. The stick models of IOX1, DNA chain 1 and DNA chain 2 are shown in yellow, cyan, and blue, respectively. Hydrogen bond, hydrophobic, and electrostatic interactions are shown as green, pink, and orange, respectively. (**D**) Close-up view after 90° rotation showing a different angle view. (**E**) The mobility of supercoiled bacterial DNA following IOX1 treatment processed by relative quantitation via ImageJ software. (**F**) Enzymatic evaluation of ATPase activity of *E. coli* DNA gyrase in the presence of IOX1 from 0.5 to 10 μM compared with that of the negative control (no enzyme) and the positive control (no inhibitor).

To further understand the mode of action (MOA) of IOX1, we tested whether JIB-04, a well-known cell-permeable pan-Jumonji histone demethylase inhibitor, could also exhibit the same antimicrobial activity against *E. coli (DH5α)*. However, unlike IOX1, JIB-04 showed a negligible effect (Fig. S7). These data suggest that the antimicrobial activity of IOX1 was not due to the cell permeability of histone demethylase inhibitors but probably to the ability of IOX1 to inhibit DNA gyrase, a mechanism feature of quinolone antibiotics (2), as IOX1 is structurally similar to this family of antibiotics (Fig. 1A). Therefore, a molecular docking study was performed to investigate the binding modes between IOX1 and *E. coli* DNA gyrase. The 3D structure of *E. coli* DNA gyrase was obtained from the Protein Data Bank (http://www.rcsb.org, PDB ID: 6RKW). IOX1 was docked into the active site of *E. coli* DNA gyrase using GOLD software^20,21^. The information for the initial IOX1-binding site in *E. coli* DNA gyrase for the docking simulation was provided from the recently published 3D structure for the complex system of the ligand and DNA gyrase^22^. The best docked conformation was selected based on cluster analysis and the GOLD fitness score (Fig. 6A,B). The best cluster number from 100 trials for IOX1 was 80, and the GOLD fitness score was 43.2 (Table S1). Our molecular docking results showed that IOX1 formed hydrogen bonds with the D1-T2 and D2-T2 bases of both DNA strands (Fig. 6C,D). IOX1 also bound with the D1-A3 and D2-A3 bases of the DNA strands via hydrophobic interactions and electrostatic interactions. Interestingly, the models showed that IOX1 interacted mainly with the DNA and that the interaction with the DNA gyrase protein seemed to be very weak or minor. In conclusion, the molecular docking study clearly confirmed that IOX1 interacted mainly with the cleft of double-stranded DNA, which is bound to DNA gyrase, rather than with DNA gyrase itself. The overall binding pattern of IOX1 is in good agreement with the experimental data^22^. Furthermore, we performed a gyrase inhibition assay, and the relaxed circular PBR322 plasmid, which usually forms a supercoil structure in the presence of gyrase activity, was used as a substrate. Supercoils and relaxed plasmids were determined by agarose gel electrophoresis (Fig. 6E). Inhibition of gyrase activity increased the relaxed plasmid morphology by regulating the activity of subunit A in DNA gyrase^16^. In addition, it has been suggested that antibacterial activity is potentially demonstrated through inhibition of both subunit A and subunit B of DNA gyrase^12^; moreover, our binding test confirmed the binding of IOX1 to the whole DNA gyrase (Fig. S8A) and precisely its binding to the DNA Gyrase Subunit B (Fig. S8B). Therefore; we tried to determine whether IOX1 directly regulates the activity of DNA Gyrase Subunit B by performing a DNA gyrase ATPase binding assay. This method is known to measure the conversion of NADH to NAD^+^ by ATP hydrolysis and assess NAD^+^ production via a 340 nm spectrogram^19^. DNA gyrase ATPase activity was reduced in the presence of IOX1 compared to that in the negative control (Fig. 6F). These results demonstrate that IOX1 is involved in both the A and B subunits of DNA gyrase, inhibiting overall gyrase activity.

### IOX1 increases the bactericidal effect against carbapenem-resistant *A. baumannii*

**Figure 7:**
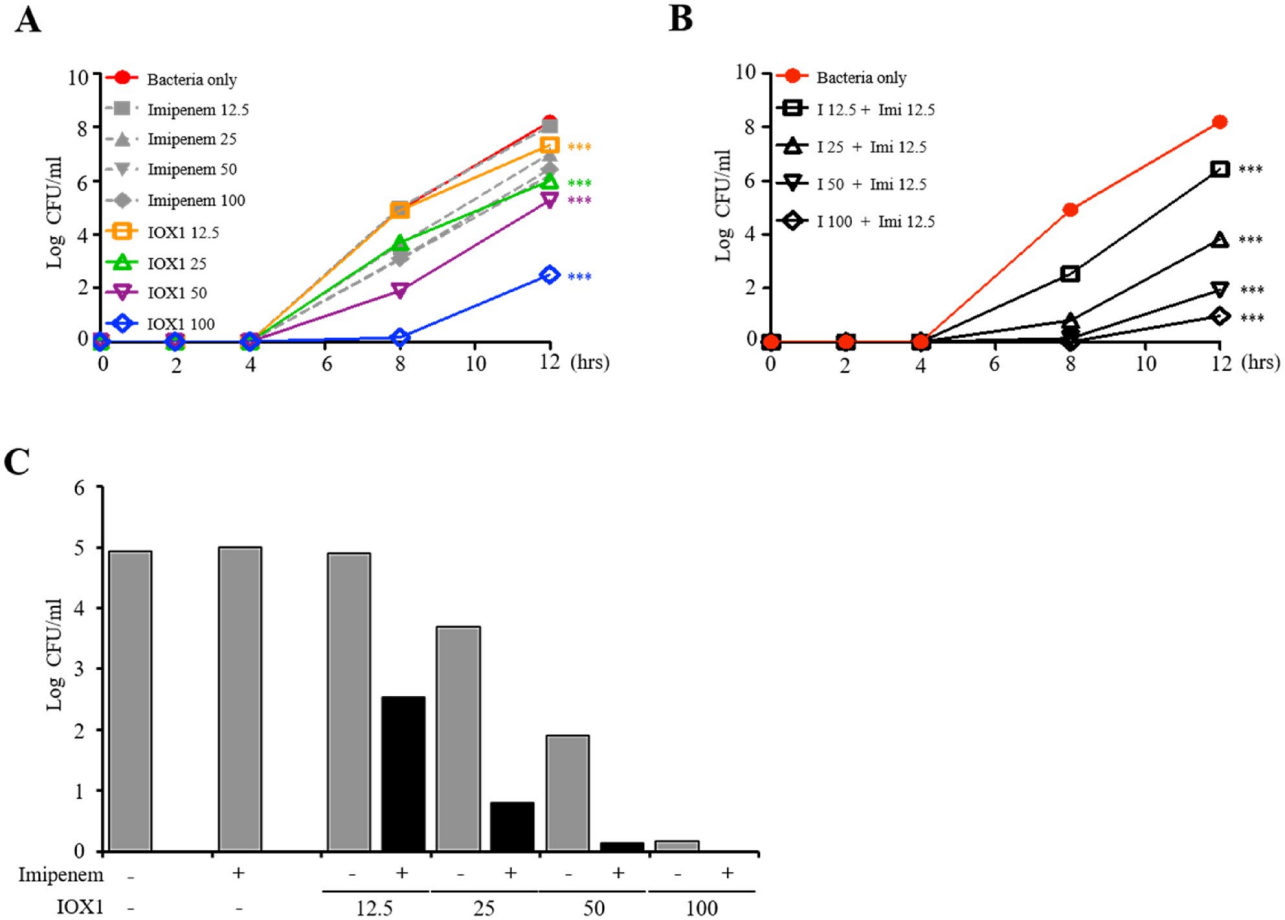
IOX1 enhances the killing of carbapenem-resistant *A. baumannii*. (**A**) Bactericidal kinetics of imipenem against resistant and susceptible CRAB 90 (4.66 × 10^4^ CFU/ml) compared to those of IOX1. (**B**) Synergistic antibacterial effect of a low concentration combination of IOX1 and imipenem on CRAB 90 (4.66 × 10^4^ CFU/ml). (**C**) The effect of IOX1 on bacterial growth in the presence or absence of imipenem at 8 h of culture.

The antimicrobial activity of IOX1 was also tested against resistant strains of bacteria. Carbapenem-resistant *A. baumannii* (CRAB) was found to proliferate in the presence of high concentrations of imipenem; in contrast, IOX1 succeeded in inhibiting bacterial growth in a concentration-dependent manner at up to 8 h (Fig. 7A). Additionally, analysis of time-kill kinetics profiles of imipenem (carbapenem antibiotic), ciprofloxacin (quinolone antibiotic) and IOX1 showed that, unlike IOX1, imipenem and ciprofloxacin exhibited no inhibitory growth effects on multidrug (carbapenem and quinolone)-resistant (MDR) *A. baumannii* (Fig. S9A–D). Interestingly, IOX1 treatment with imipenem (12.5 μg/ml) suggested a synergetic effect on CRAB growth (Fig. 7B). This observation was further demonstrated when enhanced antimicrobial effects were observed by combining imipenem with IOX1 (12.5, 25, 50 and 100 μg/ml) (Fig. 7C). This result shows that IOX1 exerts a promising antibacterial effect on MDR bacteria.

### IOX1 antiseptic effect on a carbapenem-resistant *A. baumannii*-inoculated septic mouse model

**Figure 8:**
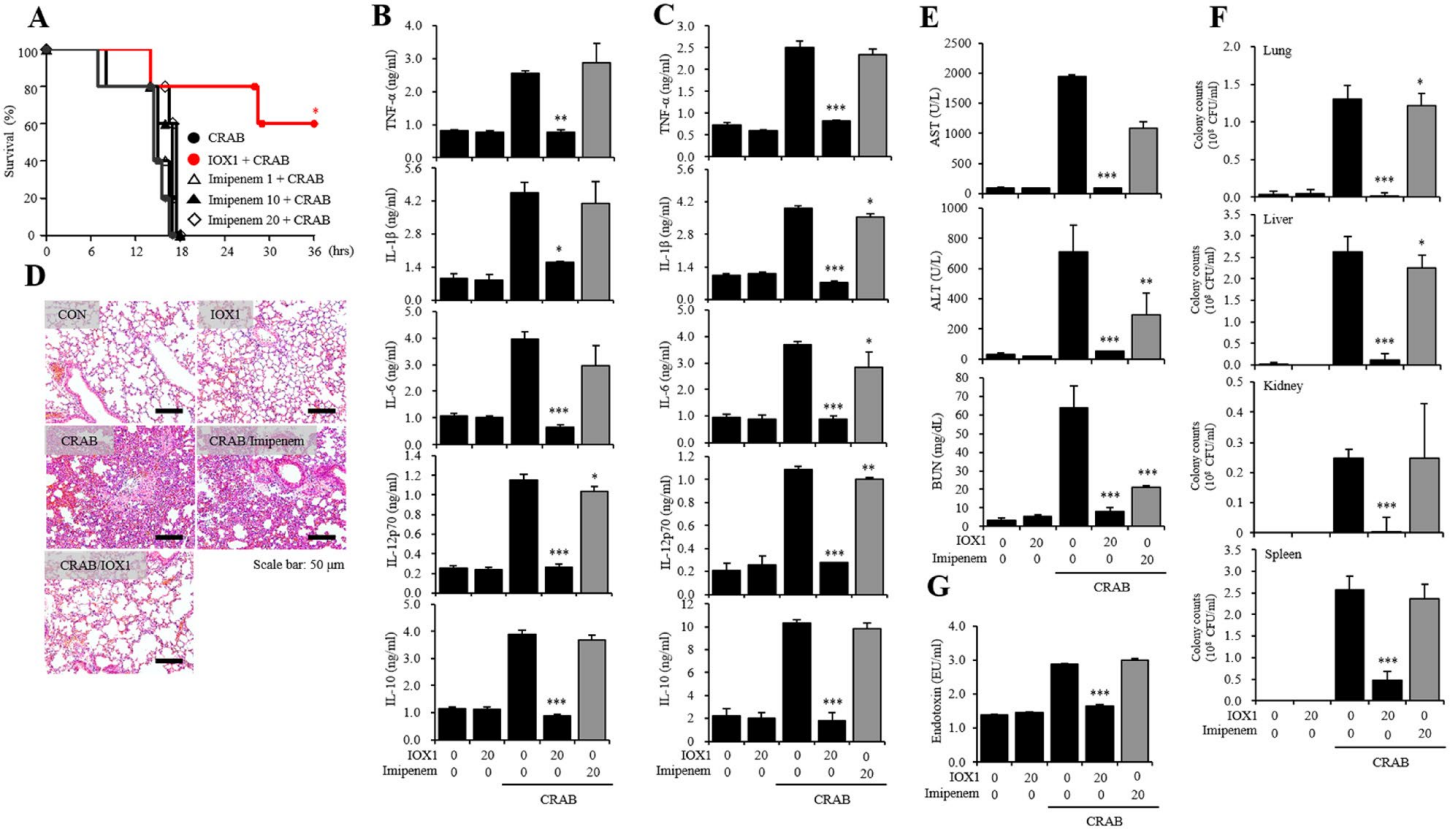
IOX1 antiseptic effect on a carbapenem resistant *A. baumannii*-inoculated septic mouse model. Six-week-old female BALB/c mice were intraperitoneally injected with IOX1 (20 mg/kg). After 30 min, the mice were i.p. injected with CRAB. (**A**) The survival rate of IOX1- and CRAB (1.4 × 10^4^ CFU/mouse)-injected mice was monitored for 48 h. (**B**) The serum of IOX1- and CRAB (7 × 10^3^ CFU/mouse)-injected mice was harvested 2 h after LPS injection. The serum levels of inflammatory cytokines (TNF-α, IL-1β, IL-6, IL-12p70 and IL-10) were measured by sandwich ELISA kits. (**C–G**) After overnight incubation, IOX1- and CRAB (7 × 10^3^ CFU/mouse)-injected mice were sacrificed for experiments. (**C**) The mouse lungs were homogenized by stainless steel beads. The lung levels of inflammatory cytokines (TNF-α, IL-1β, IL-6, IL-12p70 and IL-10) were measured by sandwich ELISA kits. (**D**) The PMN infiltrations in the lung were stained by hematoxylin and eosin based on the standard H&E staining method. (**E**) The serum levels of AST, ALT, BUN and creatinine were measured by a laboratory medicine system. (**F**) The mouse lungs, livers, kidneys and spleens were homogenized by stainless steel beads. The lysates were diluted with PBS and incubated on LB agar plates overnight. (**G**) The serum levels of endotoxin were determined by the LAL method and measured at 405 nm.

We sought to study the antiseptic effect of IOX1 in vivo*.* For this aim, we used a CRAB-inoculated septic mouse model. After 30 min of pretreatment with IOX1, mice were injected with CRAB (1.4 × 10^4^ CFU/mouse). Within 18 h, the survival rates of mice injected solely with CRAB and mice treated with imipenem were 0%. However, IOX1 was able to increase the survival rate to 80% and to maintain it at 60% for 36 h (Fig. 8A). Additionally, unlike imipenem, which showed no inhibitory effect, the levels of inflammatory cytokines, including IL-10, TNF-α, IL-1β, IL-6 and IL-12p70, in serum (Fig. 8B) and in the lung (Fig. 8C) were reduced by IOX1 administration. IOX1 was also found to be effective in reducing lung PMN infiltration (Fig. 8D) and in attenuating organ damage, as reflected by a reduction in the levels of AST, ALT, BUN and creatinine (Fig. 8E). IOX1-mediated bacterial clearance was also investigated, and the data showed an abrogated number of remaining bacteria in the liver, lungs, kidneys and spleen (Fig. 8F). Furthermore, IOX1 reduced the endotoxin levels in mouse serum that were originally induced by CRAB inoculation (Fig. 8G). These results show the promising therapeutic effects of IOX1 on sepsis triggered by carbapenem-resistant bacteria.

## Discussion

Unlike other histone modifications, histone methylation was discovered relatively late, and study of the reversible reaction is still underway^23^. However, recent histone demethylase studies have been actively conducted in several fields; for example, Kdm6b is involved in the expression of comprehensive inflammatory genes in macrophages^24^ and Kdm4a is markedly expressed in neuroectodermal stem cells to induce tumor development^25^. The most widely used 2OG analogs N-oxalyl glycine (NOG) and pancreatic ductal adenocarcinoma (PDAC), general inhibitors of 2OG oxygenase, have poor cell permeability^26^, requiring the use of prodrug diester derivatives, whereas IOX1 can be transferred to the cytoplasm and nucleus without ester modification^27^. Herein, we confirmed that IOX1 inhibited the induction of proinflammatory cytokines in LPS-stimulated DCs and, in parallel, inhibited the activity and expression of Kdm4a and Kdm6b induced by LPS (Figs. 1 and 2). In addition, IOX1 showed significant therapeutic effects on bacterial-induced mouse sepsis models, including those with antibiotic-resistant bacteria. In particular, the bacterial count in each organ caused by bacterial infection was demonstrated to be reduced (Figs. 4F, 8F, and S3F). Similar to the other derivatives, IOX1, one of the small molecules with improved physicochemical properties among 8-hydroxyquinolines, displayed direct antimicrobial activity. Remarkably, it showed broad antimicrobial effects on *E. coli*, *Acinetobacter baumannii* (AB), double antibiotic-resistant (MDR) bacterium (quinolone and carbapenem-resistant AB), colistin-resistant AB and gram-positive bacteria, thus demonstrating excellent antimicrobial performance. We elucidated the mechanism of action that modulates the broad-spectrum antimicrobial activity of IOX1 (Fig. 6). In addition to immunosuppressive activation of IOX1 through Kdms, we found that nonquinolone antibiotics rarely inhibit bacterial DNA gyrase, resulting in bacterial antimicrobial activity and anti-inflammatory responses. As a demonstration of dual function, we presented molecular modeling based on structural analysis that can be directly linked as direct and indirect evidence (Fig. 6 and Table S1) and showed that IOX1 simultaneously binds to bacterial DNA gyrase subunit B through isothermal titration calorimetry (ITC) analysis (Fig. S8B). Its mode of action is unique in that it directly interacts with DNA gyrase to prevent its binding to DNA. This study provides the first structural explanation of the *Escherichia coli* DNA gyrase-targeting mechanism of IOX1. Developing sepsis drugs has been limited by the availability of a mouse model for drug screening, which is currently regarded as a challenge, comprehending that inflammatory conditions, such as trauma, burns, and infections, in mice are significantly different from those of the human immune system. Moreover, most studies are based on the host’s immune system; therefore, it is very likely that the compounds that show efficiency in mice will not be as effective in humans because of the variation in the immune system response and the whole physiological system between the two species. Extensive studies on the introduction of Kdm or topoisomerase inhibitors have recently been initiated, but none have been shown to simultaneously inhibit Kdm and DNA gyrase, as IOX1 does. These results demonstrate that IOX1 can be used as a broad-spectrum antibiotic, with the possibility of expanding the opportunity to develop and improve new drugs.

## Materials and methods

### Animals

Six-week-old female C57BL/6 mice (H-2 Kb and I-Ab) and 6-week-old Female BALB/c mice (20 mg) were purchased from Orient Bio (Daejeon, South Korea). All experiments were performed in accordance with relevant guidelines and regulations, and all procedures were monitored and approved by the Institutional Animal Care and Use Committee (IACUC) of Konkuk University (IACUC number: KU17044-2)^30,31^.

### Bacteria

*E. coli* DH5α (ATCC PTA-4750), *Acinetobacter baumannii* (KUMC ATCC 19606, used in vivo), *Acinetobacter baumannii* (KUMC.2015.sus, used in time kill assays) and Staphylococcus aureus USA:300 (ATCC BAA-1556) was obtained from the American Type Culture Collection (Manassas, VA, USA). The *E. coli* K1 strain RS218 (O18:K1:H7) used in the *E. coli* K1-induced sepsis mouse model was kindly gifted by Dr. Jang-Won Yoon of Kangwon National University (Gangwon, South Korea). Carbapenem-resistant *Acinetobacter baumannii* was obtained from a patient with informed consent following the approved protocol of the Institutional Review Board for Human Study at Korea University Anam Hospital (ED14326/AN14326-001). All methods were carried out in accordance with relevant guidelines and regulations. Multi drug resistant (MDR)-AB strain 15–20, 15–21, K-YYK-21 and K-YYK-22 used in the bacterial sepsis mouse model were kindly gifted by Dr. Min‐Goo Lee of Korea University College of Medicine (Seoul, South Korea)^30,31^.

### Reagents and antibodies

Recombinant mouse granulocyte–macrophage colony stimulating factor (GM-CSF) was purchased from BioLegend (San Diego, CA, USA). To maintain DCs, RPMI 1640 medium, fetal bovine serum (FBS), and penicillin–streptomycin solution were used, purchased from Biowest (Nouaille, France). *E. coli* O111:B4 LPS used in the in vitro experiments was purchased from Invivogen (San Diego, CA, USA) and *E. coli* O127:B8 LPS used for the in vivo experiments was purchased from Sigma-Aldrich (St. Louis, MO, USA). Quinolone antibiotics ciprofloxacin was purchased from Sigma-Aldrich (St. Louis, MO, USA). IOX1 and JIB-04 were purchased from Selleckchem (Houston, TX, USA). An MTT Cell viability kit was purchased from Promega (Madison, WI, USA). For western blotting, Kdm4A, H3K9me3, H3K36me3 and H3 were purchased from Abcam (Cambridge, MA, USA). Protease inhibitor cocktail Luria–Bertani (LB) broth powder and LB agar powder were both purchased from Biobasic (Amherst, NY, USA) and a LAL Endotoxin Detection Kit was purchased from Lonza (Basel, Switzerland)^30,31^.

### Generation of murine bone marrow DCs (BMDCs)

Bone marrow from the tibias and femurs was used to isolate DCs after euthanasia of C57BL/6 mice following IACUC guidelines and in compliance with ARRIVE guidelines. All methods were carried out in accordance with relevant guidelines and regulations. Red blood cells were removed from the bone marrow using red blood cell lysis buffer, leaving only progenitor cells. These progenitor cells were placed into cell culture plates with RPMI 1640 (containing 10% FBS, 1% penicillin/streptomycin, and 10 ng/ml of GM-CSF) and incubated for 6 d at 37 °C under a 5% CO2 atmosphere. After six days, precursor cells had differentiated into immature DCs. To produce mature DCs, immature DC were treated with 50 ng/ml LPS and incubated overnight at 37 °C under 5% CO_2_^30,31^.

### Cell viability assay

The BMDCs were seeded at 2 × 10^6^ cells/well in a 96-well microplate and incubated with IOX1 (10, 50, 100, 200, and 500 μM) for overnight. Cell Viability Assay is assessed the ATP present, an indicator of metabolically active cells using Luminescent cell viability kit (Promega, Madison, WI, USA). The ATP present into the cell culture supernatant was quantified using a Luminometer^30,31^.

### Cytokine enzyme-linked immunosorbent assays (ELISAs)

The levels of various pro-inflammatory cytokines, including tumor necrosis factor-α (TNF), interleukin 1β (IL-1β), interleukin 6 (IL-6), and interleukin 12p70 (IL-12p70) and anti-inflammatory cytokine, interleukin 10 (IL-10) were measured by sandwich ELISA (eBioscience, San Diego, CA, USA). Optical density at 450 nm was measured using a Sunrise Spectrophotometer (TECAN, Männedorf, Switzerland)^30,31^.

### Surface staining

The BMDCs were stained by FITC anti-CD11c antibody (Clone: N418), PE anti-CD86 antibody (Clone: GL-1), PE anti-H-2Kd/H-2Dd antibody (Clone: 34–1-2S) and PE anti-I-A/I-E PE antibody (Clone: M5/114.15.2) (BioLegend). The expressions of CD80, CD86, MHC class I and MHC class II on the BMDC were analyzed by flow cytometry (FACSCalibur, BD).

### Real time-polymerase chain reaction (RT-PCR)

Total RNA was extracted using TRIzol reagent (Thermo Fisher Scientific, San Jose, CA, USA), digested with DNase I (Biobasic, Amherst, NY, USA), and reverse transcribed using a High-Capacity cDNA Reverse Transcription Kit (Applied Biosystems, Foster City, CA, USA). Amplification of the cDNA was performed using a LightCycler 480 II (Roche, Basel, Switzerland) and LightCycler 480 SYBR Green I Master mix (Roche, Basel, Switzerland), according to the manufacturer’s recommended conditions^30,31^. The PCR primer sequences are given in Table S2.

### Detection of AST, ALT, BUN and creatinine in mouse serum

Serum levels of AST (aspartate aminotransferase), ALT (alanine aminotransferase), BUN (blood urea nitrogen) and Creatinine were analyzed using total laboratory automation (Hitachi, Japan) and TBA-200FR NEO (Toshiba, Japan) systems at the Konkuk University Medical Center^30,31^.

### Hematoxylin and Eosin (H&E) staining

Lungs were extracted from mice, fixed in 4% paraformaldehyde (PFA) solution, and dehydrated to prepare paraffin blocks. The paraffin blocks were then cut to a thickness of 5 μm to prepare tissue slides. Any remaining paraffin was removed with xylene. Tissue slides were then hydrated, stained with H&E, and dehydrated. After mounting the tissue slides, images were captured under a microscope^30,31^.

### Western blotting and immunoprecipitation (IP)

Proteins (a total of 25 μg per sample) were separated by 10% sodium dodecyl sulfate polyacrylamide gel electrophoresis (SDS-PAGE) and transferred to polyvinylidene fluoride membranes. After blocking the membranes with 5% skim milk in Tris-buffered saline containing 0.05% Tween-20 (TBS-T), they were incubated with the indicated antibodies. After washing with TBS-T, the membranes were incubated with a secondary antibody conjugated to horseradish peroxidase and visualized using an enhanced chemiluminescence kit (Merck, Darmstadt, Germany). For immunoprecipitations (IP), the corresponding Ab was added to 1 ml each of the cellular extracts and incubated at 4 °C for 1 h on a rotator. Fifty microliters of a 50% slurry of prewashed protein A-agarose beads was then added to each sample, followed by incubation for an additional 12 h at 4 °C. The samples were washed four times in lysis buffer and subjected to Western blot analyses as described above^30,31^.

### Determination of bacterial counts in organ tissues

At the time of sacrifice, the lungs, liver, and kidneys were removed aseptically and placed separately in 1 ml of sterile PBS. The tissues were then homogenized on ice using a tissue homogenizer under a vented hood. The lung, liver, and kidney homogenates were diluted with PBS to 1:1000. After plating 10 μL of each diluted sample onto LB agar, the plates were then incubated at 37 °C for 24 h. The numbers of colonies were then counted and used to assess the relative abundances of bacteria^30,31^.

### Detection of endotoxin in mouse serum

The levels of endotoxin in mouse sera were measured using a LAL chromogenic end point assay (Lonza Group Ltd., Allendale, NJ, USA), according to the manufacturer’s recommendations. Mouse sera were diluted tenfold in endotoxin-free PBS before the assay. After subtracting background levels, the results were calculated relative to an E. coli endotoxin standard provided with the assay kit. The relative amounts of endotoxin in each sample are expressed as EU/ml^30,31^.

### LPS-induced mouse endotoxemia model

The 6-week-old female BALB/c mice were intraperitoneally injected 20 mg/kg of IOX1 (Sellectchem). For survival rate analysis, after the 30 min, IOX1-injected mice were i.p. injected 20 mg/kg of LPS (*E. coli* O127:B8, Sigma-Aldrich, St. Louis, MO) (5 mice/group). The survival rate was observed for 48 h. For other analysis, After the 2 h, IOX1-injected mice were i.p. injected 10 mg/kg of LPS (5 mice/group). After the 2 h, the serum was harvested for pro-inflammatory cytokine ELISA. Next day, these mice were sacrificed depending on animal ethics. The half of the lung was fixed by paraformaldehyde. And then, these lungs were sliced for making tissue slide and that slides were stained by hematoxylin and eosin. The other side of lung was homogenized using a Bullet Blender homogenizer (Next Advance, NY, USA). The pro-inflammatory cytokines in lung were measured by ELISA. The AST, ALT, BUN and creatinine levels in the serum were measured using total laboratory automation (Hitachi, Japan) and TBA-200FR NEO (Toshiba, Japan) systems. The study was carried out in compliance with the ARRIVE guidelines.

### *A. baumannii (*KUMC ATCC 19606)-induced mouse sepsis model

The 6-week-old female BALB/c mice were intraperitoneally injected 20 mg/kg of IOX1 (Selleckchem). For survival rate analysis, after the 30 min, IOX1-injected mice were i.p. injected 1.9 × 10^4^ CFU/mouse of *A. baumannii* (5 mice/group). The survival rate was observed for 36 h. For other analysis, after 30 min, IOX1-injected mice were i.p. injected 9.5 × 10^3^ CFU/mouse of *A. baumannii* (5 mice/group). After the 2 h, the serum was harvested for pro-inflammatory cytokine ELISA. Next day, these mice were sacrificed depending on animal ethics. The half of the lung was fixed by paraformaldehyde. And then, these lungs were sliced for making tissue slide and that slides were stained by hematoxylin and eosin. The other side of lung, liver, kidneys and spleens were homogenized using a Bullet Blender homogenizer (Next Advance, NY, USA). The pro-inflammatory cytokines in lung lysates were measured by ELISA. The remained bacteria in tissue lysates were diluted by PBS and incubated at LB agar plate during overnight. The AST, ALT, BUN and creatinine levels in the serum were measured using total laboratory automation (Hitachi, Japan) and TBA-200FR NEO (Toshiba, Japan) systems. The levels of serum endotoxin were conducted by limulus amebocytes lysate (LAL) assay method and the color reaction were measured at 405 nm. The study was carried out in compliance with the ARRIVE guidelines.

### Carbapenem-resistant *A. baumannii* (CRAB)-induced mouse sepsis model

The 6-week-old female BALB/c mice were intraperitoneally injected 20 mg/kg of IOX1 (Selleckchem). For survival rate analysis, after the 30 min, IOX1-injected mice were i.p. injected 1.4 × 10^4^ CFU/mouse of CRAB (5 mice/group). The survival rate was observed for 48 h. For other analysis, after the 30 min, IOX1-injected mice were i.p. injected 7 × 10^3^ CFU/mouse of CRAB (5 mice/group). After the 2 h, the serum was harvested for pro-inflammatory cytokine ELISA. Next day, these mice were sacrificed depending on animal ethics. The half of the lung was fixed by paraformaldehyde. And then, these lungs were sliced for making tissue slide and that slides were stained by hematoxylin and eosin. The other side of lung, liver, kidneys and spleens were homogenized using a Bullet Blender homogenizer (Next Advance, NY, USA). The pro-inflammatory cytokines in lung lysates were measured by ELISA. The remained bacteria in tissue lysates were diluted by PBS and incubated at LB agar plate during overnight. The AST, ALT, BUN and creatinine levels in the serum were measured using total laboratory automation (Hitachi, Japan) and TBA-200FR NEO (Toshiba, Japan) systems. The levels of serum endotoxin were conducted by limulus amebocytes lysate (LAL) assay method and the color reaction were measured at 405 nm. The study was carried out in compliance with the ARRIVE guidelines.

### Time-kill kinetics assay

The bactericidal activities of IOX1 were performed using a modified time-kill kinetics assay^28^. *DH5α* (4.03 × 10^4^ CFU/ml), *A. baumannii* KUMC.2015.sus (4.3 × 10^4^ CFU/ml), CRAB (4.66 × 10^4^ CFU/ml), *MDR-A.baumannii AB 15–20* (1.7 × 10^4^ CFU/ml), 15–21 (1.5 × 10^4^ CFU/ml), K-YYK-21 (1.8 × 10^4^ CFU/ml), K-YYK-22 (3.1 × 10^4^ CFU/ml), *S. aureus* (9.0 × 10^4^ CFU/ml) and Colistin-resistant AB 2 (8.1 × 10^4^ CFU/ml) were incubated with IOX1 at different concentration (0, 12.5, 25, 50, 100 μg/ml), in MH broth at 37 °C. Imipenem and quinolone were used as positive control, and the assay was performed in triplicate. 10 μl of bacterial suspensions was removed at various time intervals (0, 2, 4, 6, 8, and 12 h), serially diluted in MH broth and plated onto LB agar overnight at 37 °C in the presence of 5% CO_2_ to obtain viable colonies. Amp was used as positive control and the assay was performed in triplicate.

### Molecular docking study

The molecular docking was performed by GOLD 5.2 (Genetic Optimization for Ligand Docking) and the standard function *GOLD Score*was used for scoring^26,27^. Discovery Studio 2018 (DS) was used for preparation of SD file for the ligand IOX1 to utilize GOLD. All the active site residues within 15 Å radius spheres of the center were included for the calculation. Other parameters set as their default and the number of GOLD runs set to 100. Top-ranked docking conformation was defined on the largest GOLD fitness score value and clustering processed in keeping with RMSD.

### Agarose gel DNA gyrase assays

Conditions to measure the supercoiling of a relaxed, closed-circular plasmid substrate and cleavage complex stabilization in agarose gels were adapted from the literature and carried out as described previously (29).

### Enzymatic assays

The DNA gyrase ATPase linked assay was performed using an *E. coli* gyrase ATPase linked assay kit according to the manufacturer’s instructions (Insiparlis cod. ATPG001). Reaction mixtures (100 μl) contained 40 mM HEPES–KOH (pH 7.6), 10 mM magnesium acetate, 10 mM DTT, 2 mM ATP, 500 mM potassium glutamate, 0.05 mg/ml albumin, 3 μg of relaxed pBR322 DNA, 80 mM PEP, stock pyruvate kinase/lactate dehydrogenase (reported concentration in the manufacturer’s instructions), 20 mM NADH, 50 nM DNA gyrase, and either 50, 25, 10, or 5 μM UVI5008 dissolved in 10% DMSO or 30 μM ciprofloxacin dissolved in 0.01 N HCl. The negative and positive controls were represented by the absence and presence of the enzyme in 10% DMSO, respectively. Absorbance at 340 nm was measured for 10 min at 25 °C. The reaction began after the addition of 30 mM ATP. Absorbance at 340 nm was then monitored for 60 min at 25 °C^30,31^.

### Statistical analysis

All experiments were repeated at least three times with consistent results. Unless otherwise stated, data are expressed as means ± SEM. A Student's t-test was performed to compare experimental groups and controls and a Tukey's multiple comparison test using Prism v3.0 (GraphPad Software, La Jolla, CA, USA) was used to compare multiple groups. Kaplan–Meier curves for survival rates were analyzed using a log rank test. The threshold of statistical significance was set at P < 0.05^30,31^.

## Supplementary Information

Supplementary Information
