# Supplementary material for: IOX1 activity as sepsis therapy and an antibiotic against multidrug-resistant bacteria
Source: Sci Rep. 2021 Feb 3;11:2942. doi: 10.1038/s41598-021-82377-z (PMC7858575; doi:10.1038/s41598-021-82377-z)
Supplement: Supplementary file 1 — Supplementary Information [file 41598_2021_82377_MOESM1_ESM.docx]

**IOX1 activity as sepsis therapy and an antibiotic against multidrug-resistant bacteria**

Su Jin Lee^1,*^, Jueng Soo You^2,*^, Amal Gharbi^3,*^, Yong Joo Kim^3^, Mi Suk Lee^3^, Dong Hwan Kim^4^, Keun Woo Lee^4^, In Duk Jung^3,#^, Yeong Min Park^1,3,^

^1^Department of Immunology, Laboratory of Dendritic Cell Differentiation & Regulation, School of Medicine, Konkuk University, Chungju 380-701, Seoul, South Korea. ^2^Department of Biochemistry, School of Medicine, Konkuk University, Chungju 380-701, Seoul, South Korea. ^3^Dandi Bioscience Inc, 6th floor of Real Company, 66, Acha San-ro, Seongdong-gu, Seoul, South Korea. ^4^Division of Life Science, Division of Applied Life Science (BK21 Plus), Research Institute of Natural Science (RINS), Gyeongsang National University (GNU), 501 Jinju-daero, Jinju, 52828, Republic of Korea

^*^These authors contributed equally to this work

^#^Corresponding authors: In Duk Jung, Ph.D. Dandi Bioscience Inc, 6th floor of Real Company, 66, Acha San-ro, Seongdong-gu, Seoul, South Korea. Phone : +82-70-5101-0738 ; Fax : +82-70-8280-0738 ; E-mail : jungid@dandibio.com & Yeong-Min Park, M.D., Ph,D. Department of Immunology, Laboratory of Dendritic Cell Differentiation & Regulation, School of Medicine, Konkuk University, Chungju 380-701, Seoul, South Korea. Phone : +82-2-2049-6330 ; Fax : +82-2-2049-6192 ; E-mail : [immun3023@kku.ac.kr](mailto:immun3023@kku.ac.kr)

**Supporting Information**

**
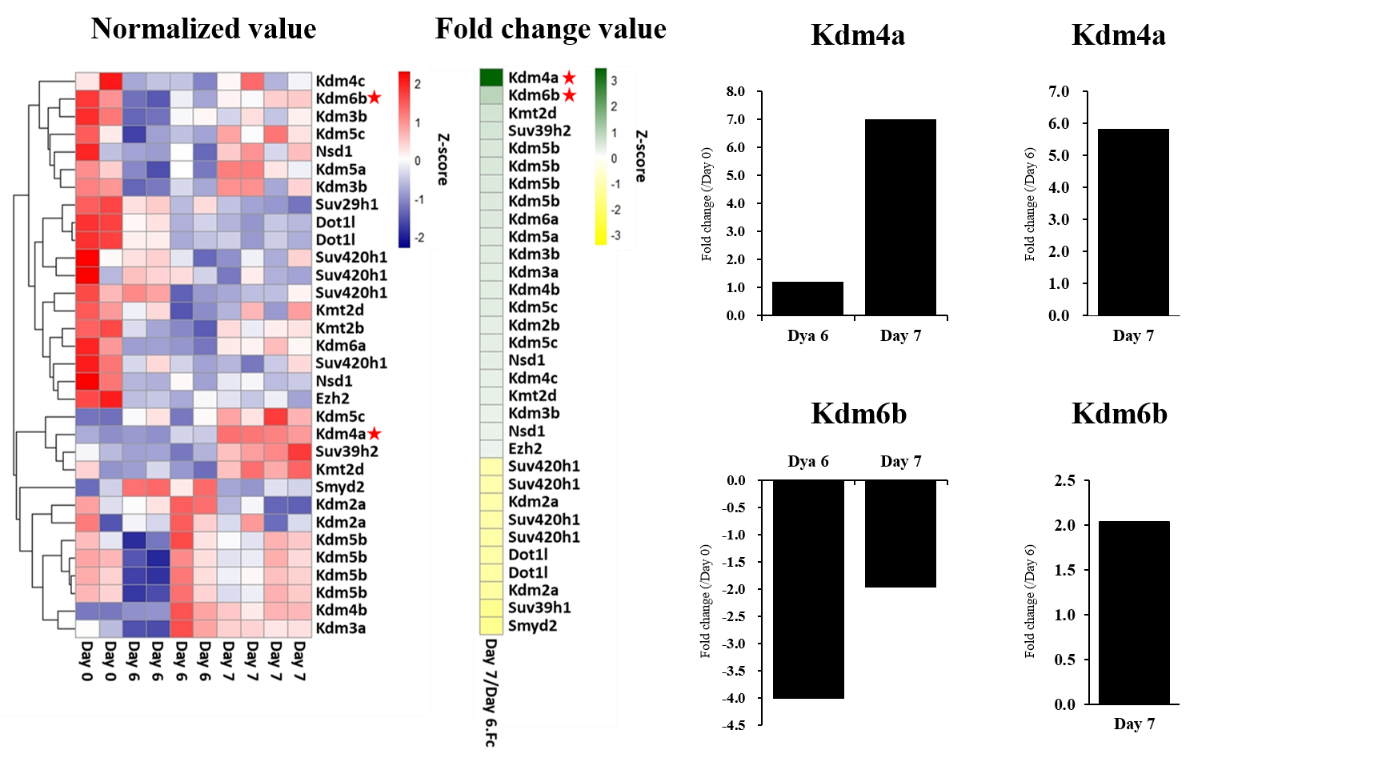
**

**Fig. S1.** The expression of Kdm4a and kdm6b is induced during maturation of DCs induced by LPS. (a) Heatmap showing expression levels for samples of LPS-induced DCs on days 0, 6 and 7 for genes encoding significant histone demethylases. (b) Graph showing the levels of specifically Kdm4a and Kdm6b associated with LPS-induced DC maturation.

**
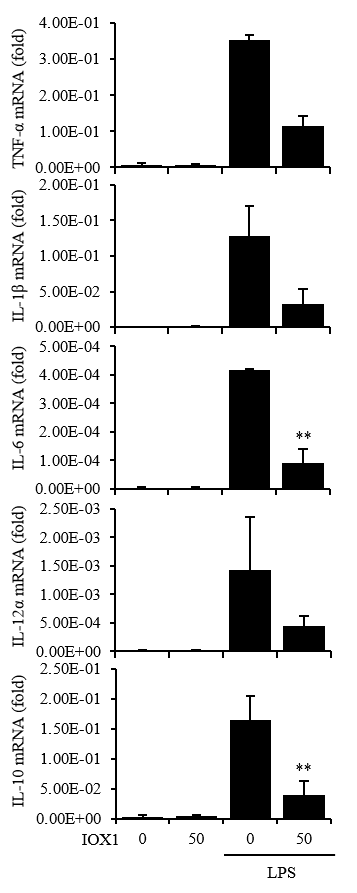
**

**Fig. S2.** RNA extraction and reverse transcription-quantitative polymerase chain reaction (RT-qPCR) analysis. Total RNA was extracted using TRIzol reagent. The RNA samples were adjusted to the same concentration and reverse transcribed using a high‑capacity cDNA reverse transcription kit (Applied Biosystems; Thermo Fisher Scientific, Inc.; cat. no. 4368813). Synthesized cDNA was generated using a Cycler® 480 II with LightCycler® 480 SYBR Green I master mix (Roche Diagnostics, Basel, Switzerland; cat. no. 04887352001) according to the manufacturer's protocols. The RT-qPCR primer sequences used were as follows: Tnf-α, forward, 5'-TCGTAGCAAACCACCAAGTG-3' and reverse, 5'-AGATAGCAAATCGGCTGACG-3'; Il-1β, forward, 5'-GACCTTCCAGGATGAGGACA-3' and reverse, 5'-TCCATTGAGGTGGAGAGCTT-3'; Il-6, forward, 5'-CTTGGGACTGATGCTGGTGA-3' and reverse, 5'-TGCAAGTGCATCATCGTTGT-3'; Il-10, forward, 5'-GGTTGCCAAGCCTTATCGGA-3' and reverse, 5'-ACCTGCTCCACTGCCTTGCT-3'; Il-12α, forward, 5'-CCAGGGTCATTCCAGTCTCT-3' and reverse, 5'-TCTTCAATGTGCTGGTTTGG-3'; and β-actin, forward, 5'-AAGTGTGACGTTGACATCCG-3' and reverse, 5'-GATCCACATCTGCTGGAAGG-3'. RT-qPCR analysis was performed with an initial denaturation step of 5 minutes at 95°C, followed by 45 cycles at 95°C for 10 sec, 60°C for 10 sec, and 72°C for 10 sec. Quantification of RNA values was determined automatically using the LightCycler® 480 program (Roche Diagnostics).

**
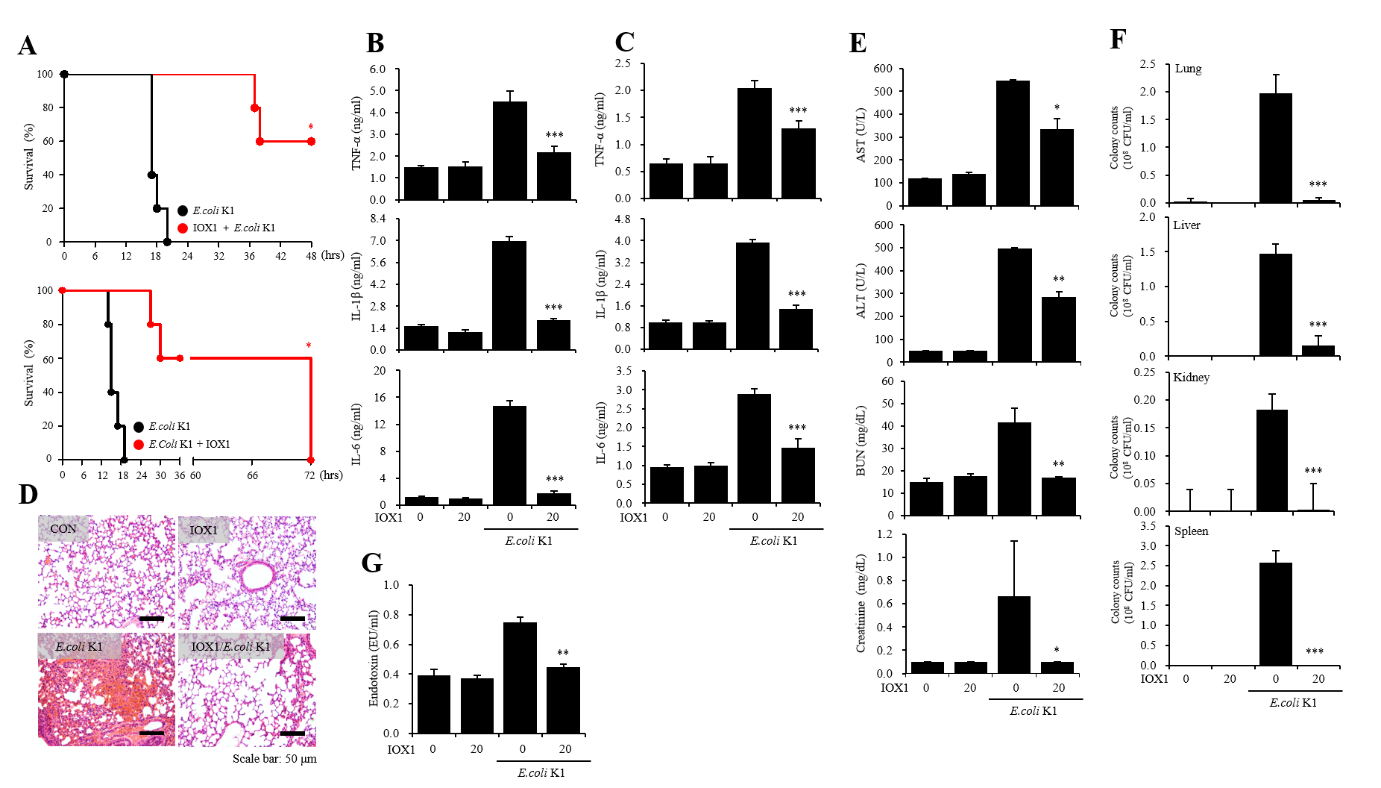
**

**Fig. S3.** IOX1 antiseptic effect on an *E. coli* K1-inoculated septic mouse model**.** Six-week-old female BALB/c mice were intraperitoneally injected with IOX1 (20 mg/kg). (A) The survival rate of mice treated with IOX1 after 30 minutes pre-inoculation or post-inoculation of *E. coli* (3.9 × 10^6^ CFU/mouse) was monitored for 48 and 72 hrs. (B) The serum levels of proinflammatory cytokines (TNF-α, IL-1β, and IL-6) were measured by sandwich ELISA kits. (C-G) After overnight incubation, IOX1- and *E. coli* (1.3 × 10^6^ CFU/mouse)-injected mice were sacrificed for experiments. (C) The mouse lungs were homogenized by stainless steel beads. The lung levels of proinflammatory cytokines (TNF-α, IL-1β, and IL-6) were measured by sandwich ELISA kits. (D) The PMN infiltrations in the lung were stained by hematoxylin and eosin based on the standard H&E staining method. (E) The serum levels of AST, ALT, BUN and creatinine were measured by a ​laboratory medicine system. (F) The mouse lungs, livers, kidneys and spleens were homogenized by stainless steel beads. The lysates were diluted with PBS and incubated on LB agar plates overnight. (G) The serum levels of endotoxin were determined by the LAL method and measured at 405 nm.


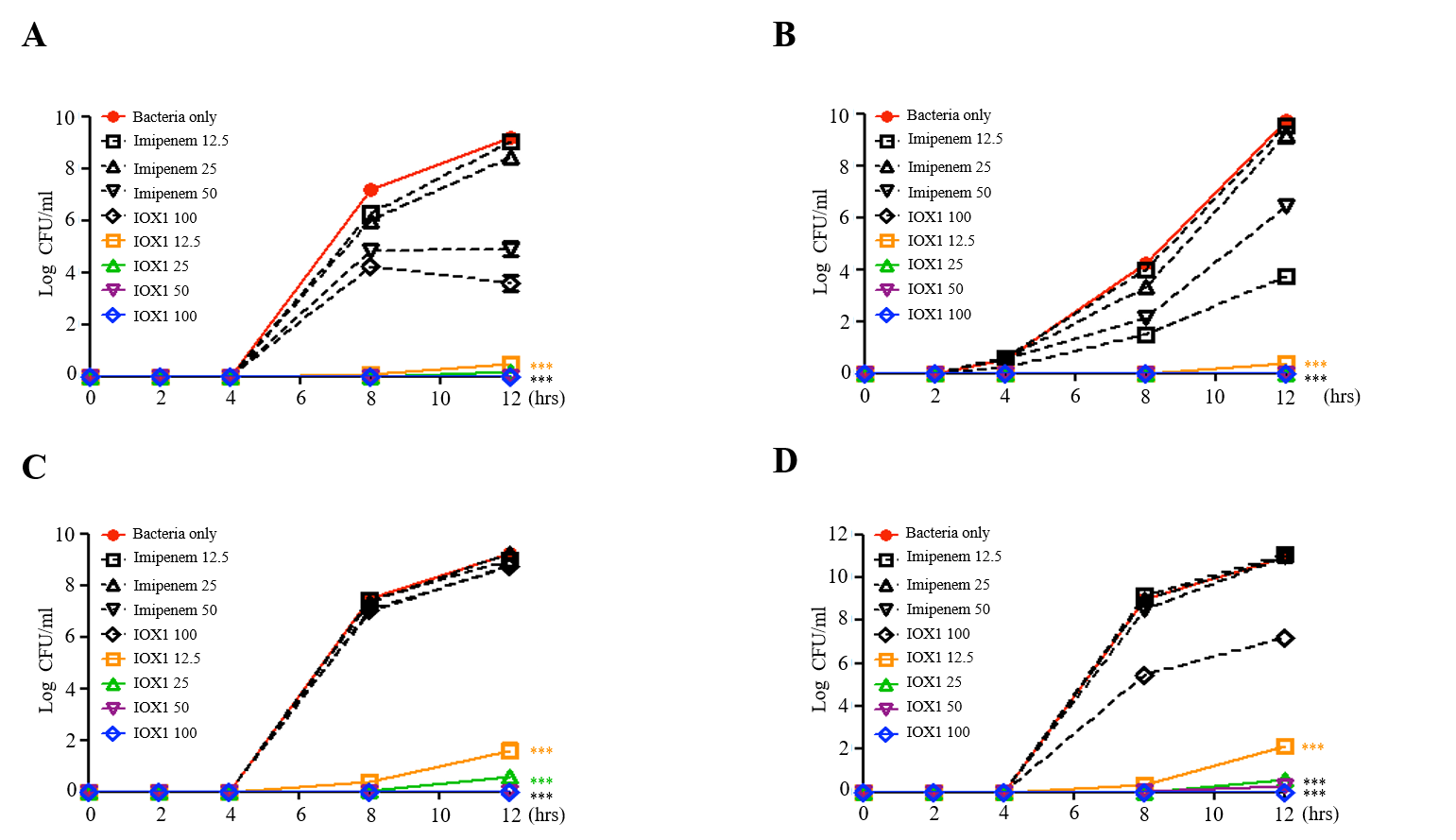


**Fig. S4.** IOX1 enhances the killing of different types of gram-negative bacteria. (A) Bactericidal kinetics of imipenem and IOX1 against *S.enteritidis* (ATCC 13076; 6.1×10^4^ CFU/ml). (B) Bactericidal kinetics of imipenem and IOX1 against *S.typhimurium* (ATCC 53648; 7.4×10^4^ CFU/ml). (C) Bactericidal kinetics of imipenem and IOX1 against *K.pneumoniae* (ATCC 13883; 5.5×10^4^ CFU/ml). (D) Bactericidal kinetics of imipenem and IOX1 against *P.aeruginosa PAO1* (ATCC 47085D-5; 5.32×10^4^ CFU/ml).

**
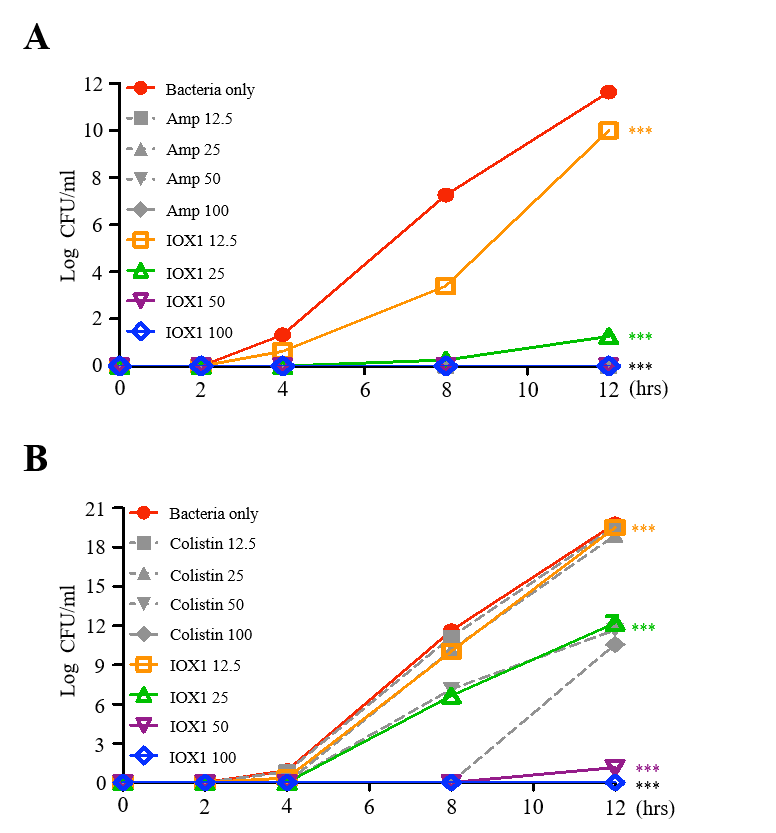
**

**Fig. S5.** IOX1 Enhances the killing of gram-positive bacteria and colistin-resistant *A. baumannii*. (A) Bactericidal kinetics of IOX1 against *S. aureus* (9.0×10^4^ CFU/ml). (B) Bactericidal kinetics of IOX1 against colistin-resistant AB 2 (8.1×10^4^ CFU/ml).


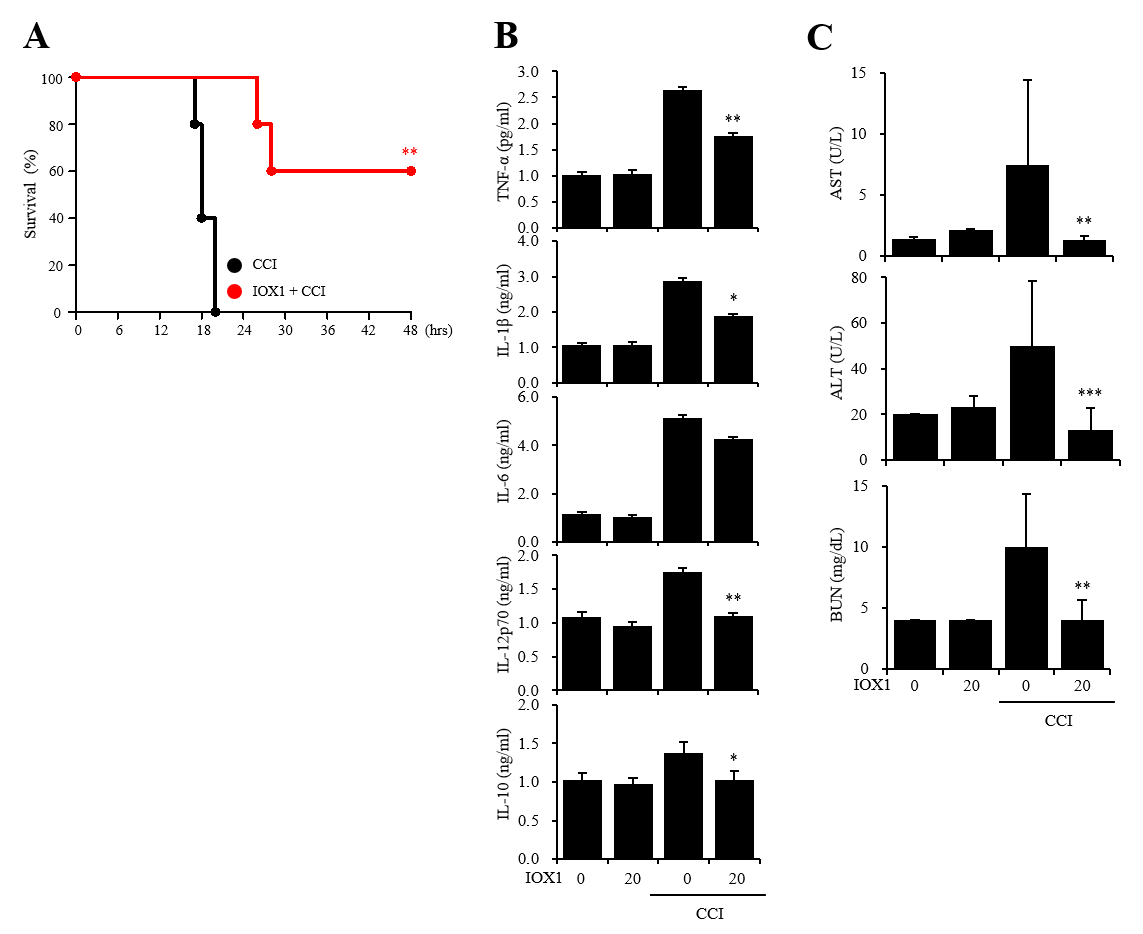


**Fig. S6.** IOX1 antiseptic effect on a CCI-induced sepsis mouse model. Six-week-old female BALB/c mice were intraperitoneally injected with IOX1 (20 mg/kg). After 30 min, the mice were i.p. injected with cecal contents. (A) The survival rate of IOX1- and 40 mg of cecal contents-injected mice was monitored for 72 h. (B) The levels of proinflammatory cytokines (TNF-α, IL-1, IL-6, IL-12p70 and IL-10) in the serum were measured using a sandwich ELISA kit. (C) The levels of AST, ALT, BUN and creatinine in the serum were measured by a ​laboratory medicine system.


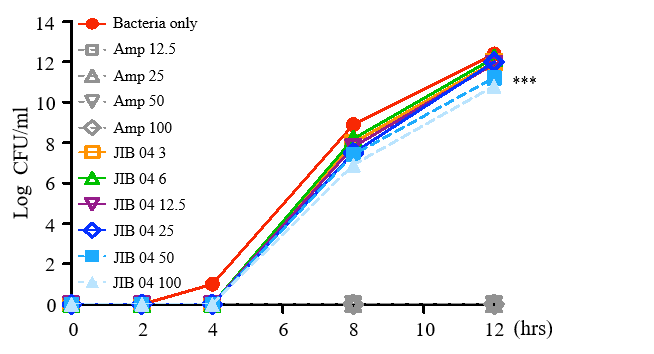


**Fig. S7.** Comparison of the antibiotic ampicillin and Jumonji histone demethylase inhibitor JIB 04 activities against *E. coli* *DH5α* bacteria (4.03×10^4^ CFU/ml).

**
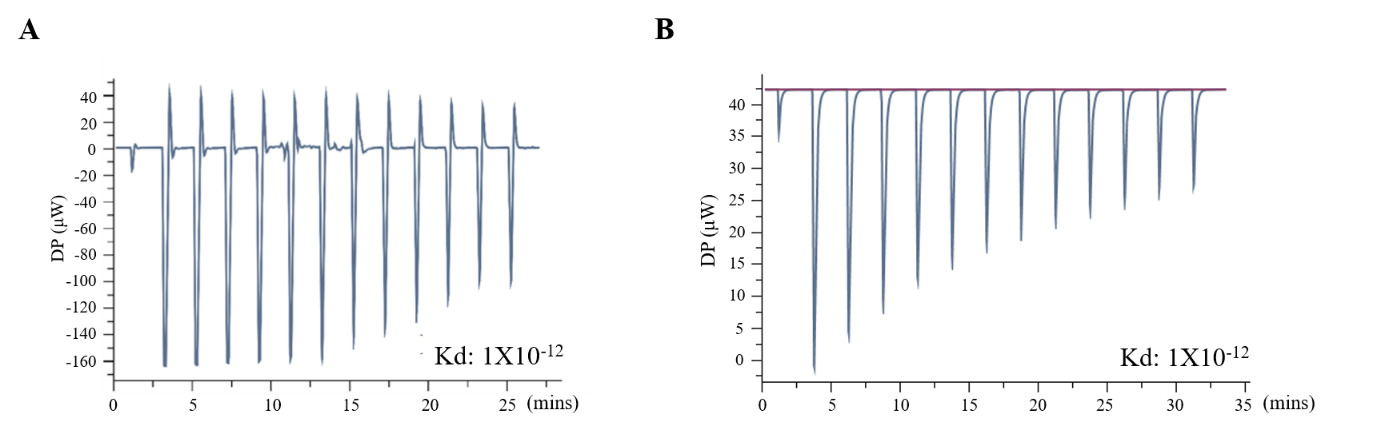
**

**Fig. S8.** IOX1 and DNA Gyrase binding. (A) IOX1 and whole DNA Gyrase binding affinity, and (B) IOX1 and DNA Gyrase Subunit B (GyrB) binding affinity demonstrated with ITC.


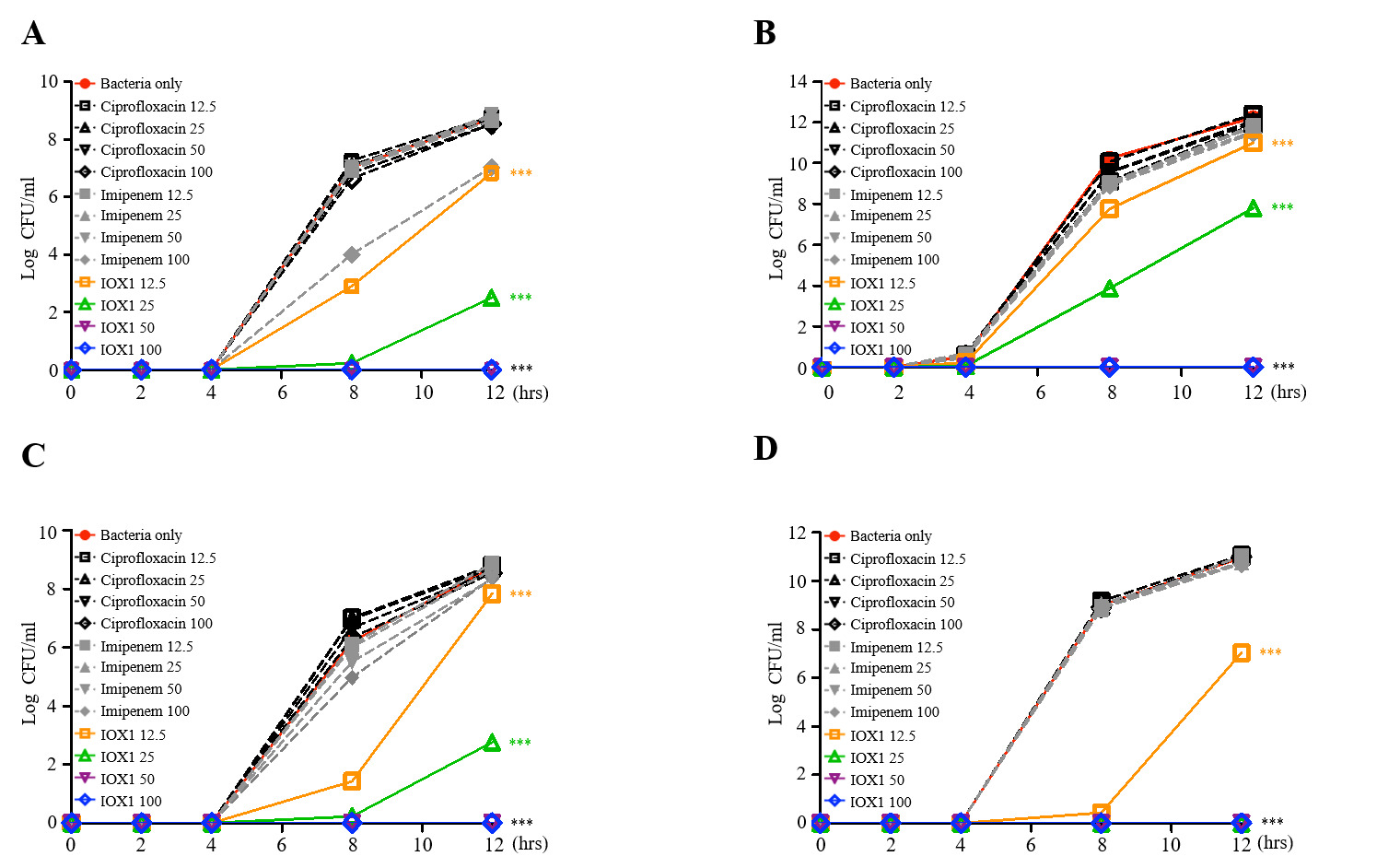


**Fig. S9.** IOX1 enhances the killing of multidrug-resistant *A. baumannii*. (A) Bactericidal kinetics of imipenem and IOX1 against quinolone-resistant *MDR-A. baumannii AB 15-20* (1.7×10^4^ CFU/ml). (B) Bactericidal kinetics of imipenem and IOX1 against quinolone-resistant *MDR-A. baumannii AB 15-21* (1.5×10^4^ CFU/ml). (C) Bactericidal kinetics of imipenem and IOX1 against quinolone-resistant *MDR-A. baumannii AB K-YYK-21* (1.8×10^4^ CFU/ml). (D) Bactericidal kinetics of imipenem and IOX1 against quinolone-resistant *MDR-A. baumannii AB K-YYK-22* (3.1×10^4^ CFU/ml).

* In vitro – KDM4A * In vitro – H3


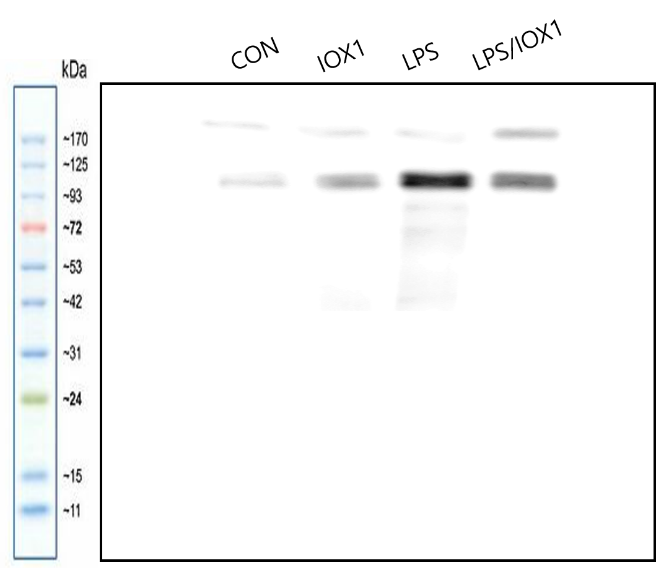

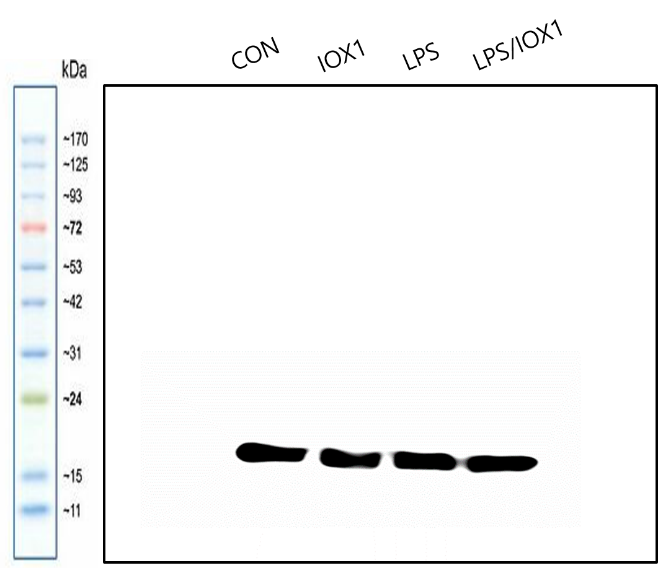


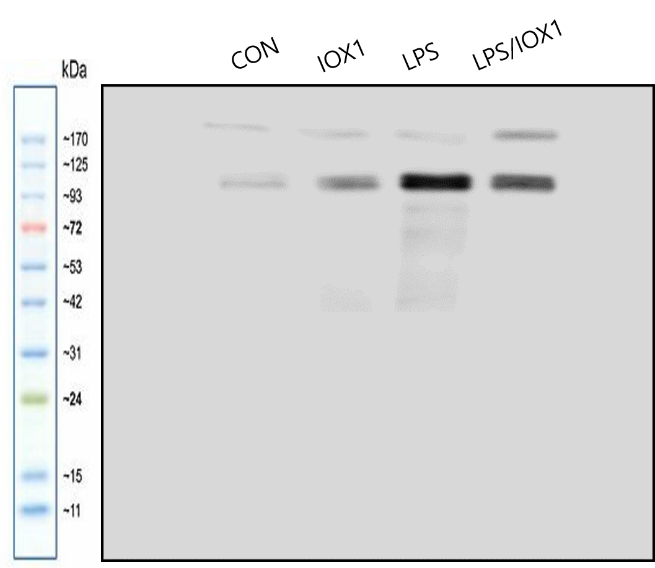

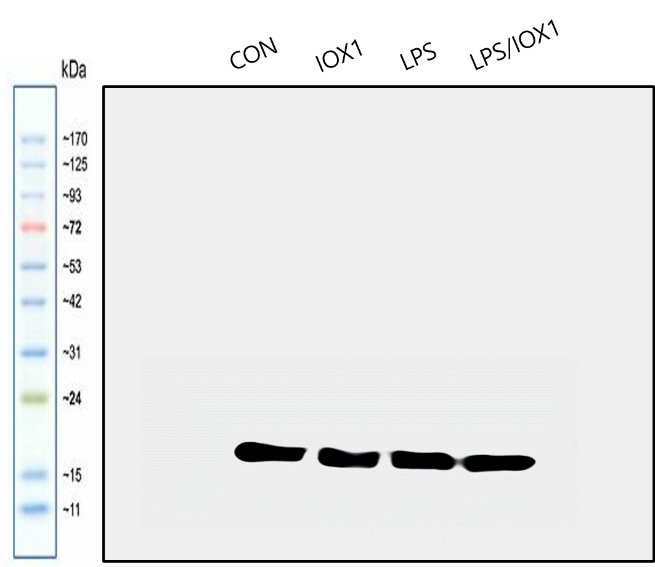


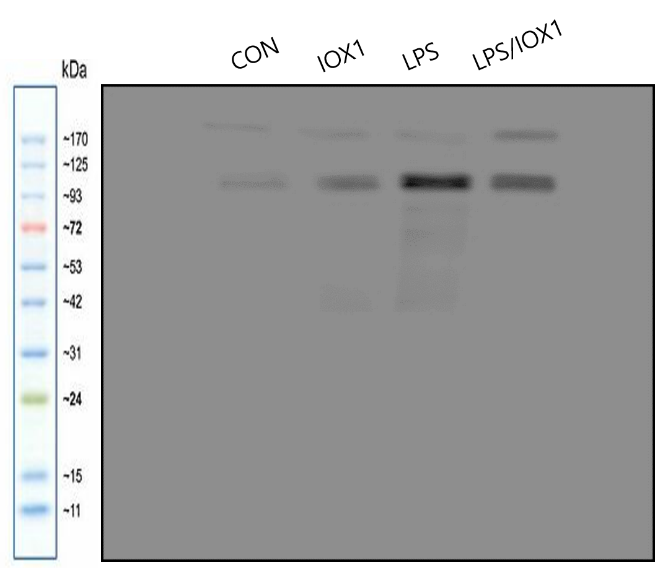

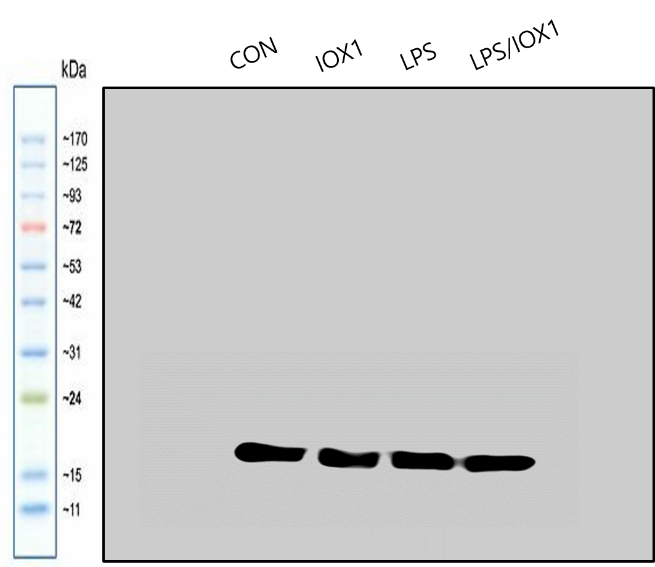


**Fig. S10.** Full-length blots of figure 2A

* In vitro – H3K9me3 * In vitro – H3K36me3


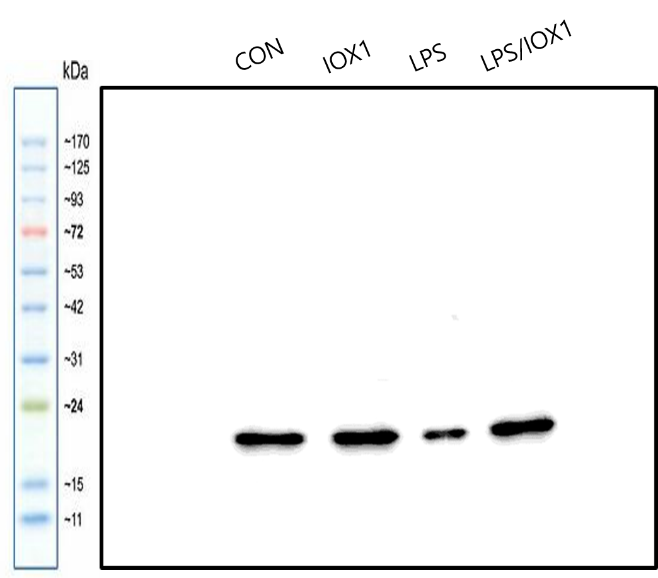

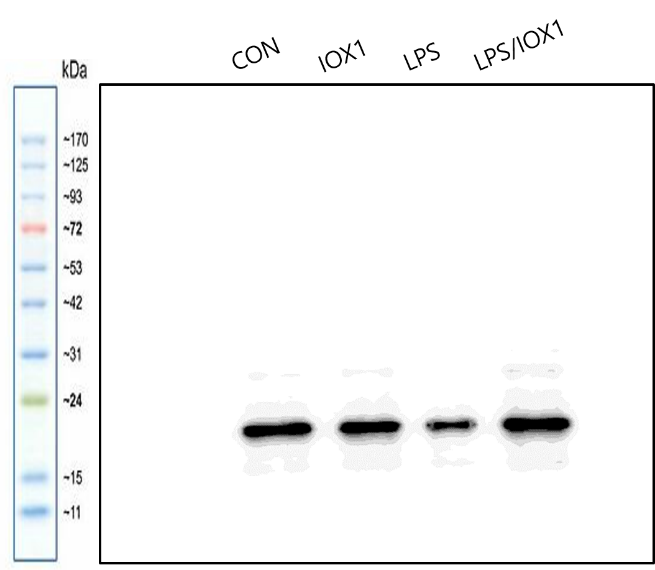


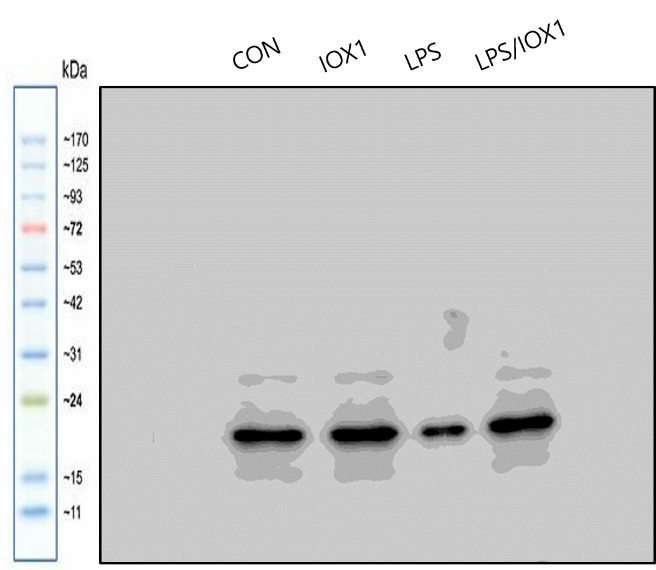

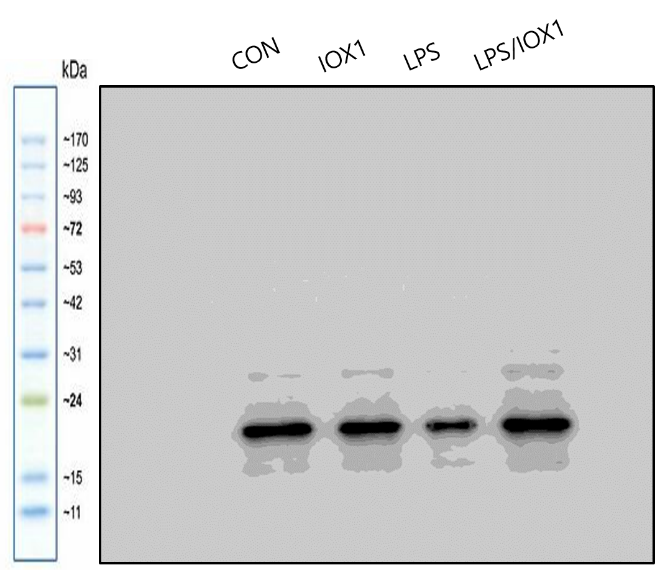


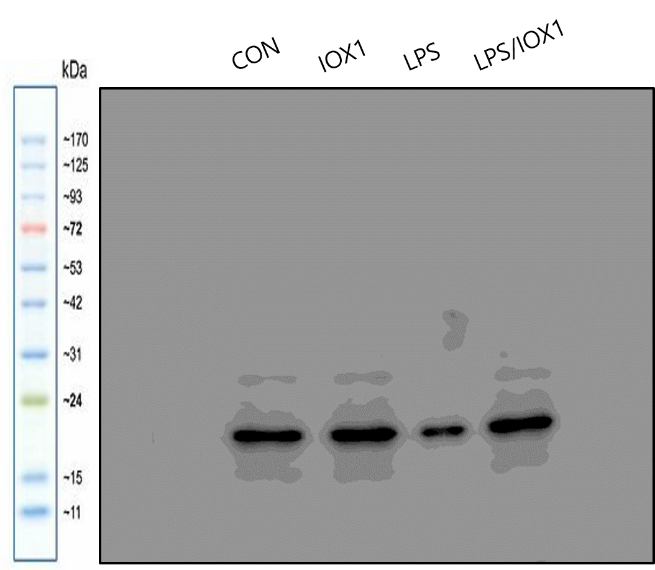

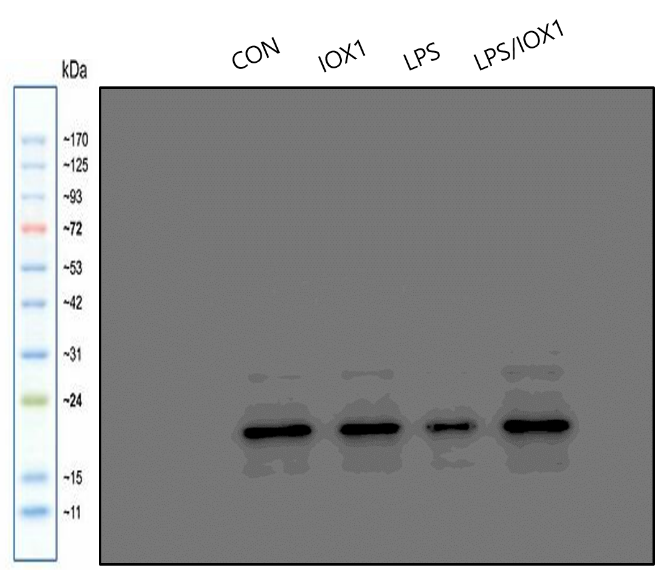


* In vitro – H3


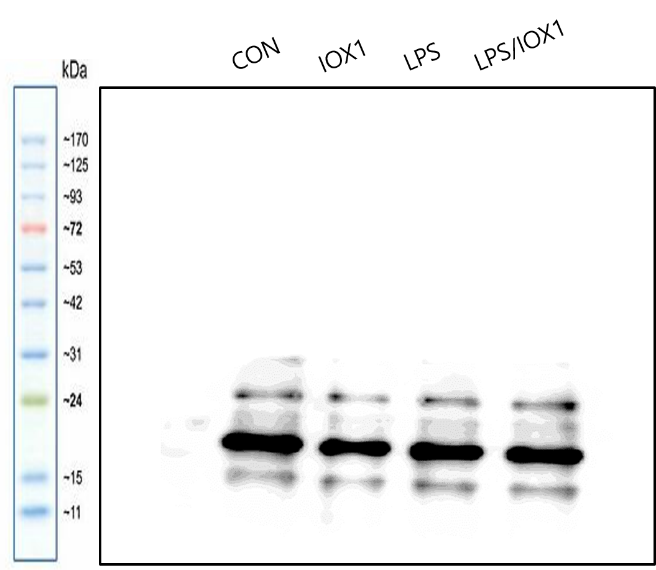


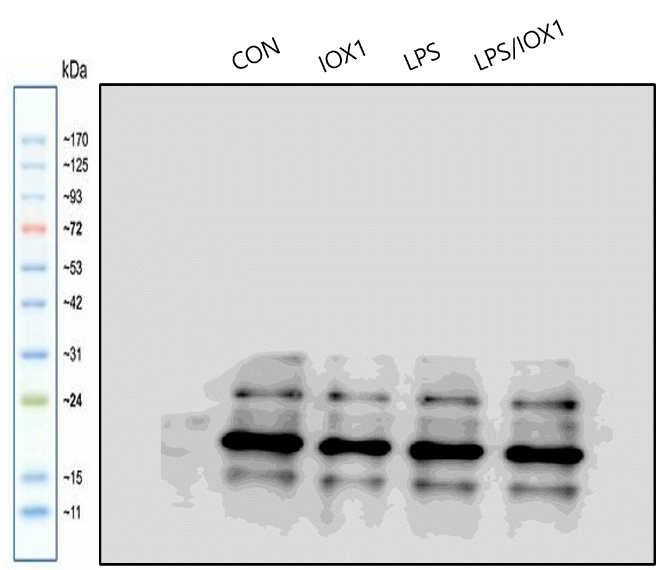


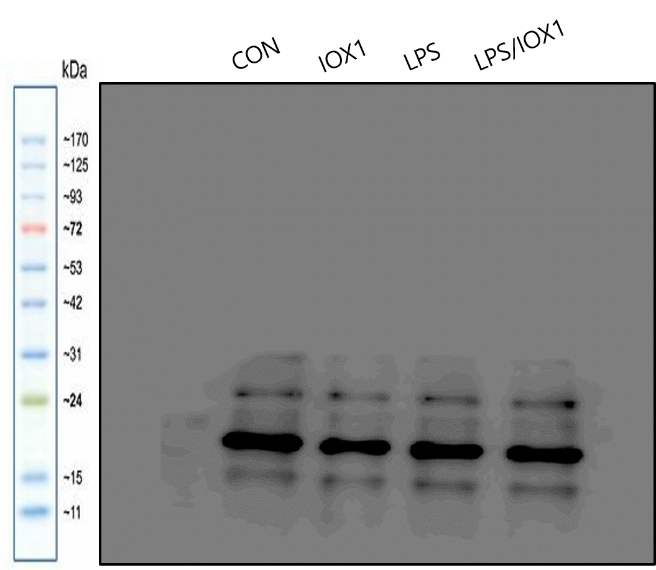


**Fig. S11.** Full-length blots of figure 2B

* In vivo – KDM4A * In vivo – H3


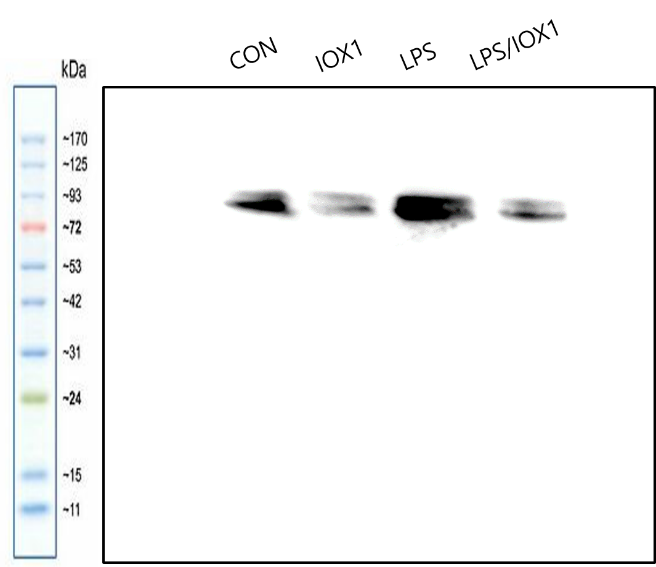

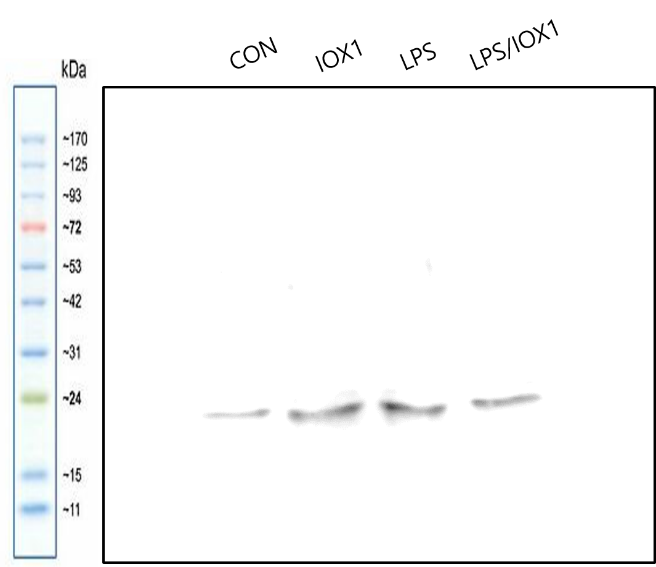


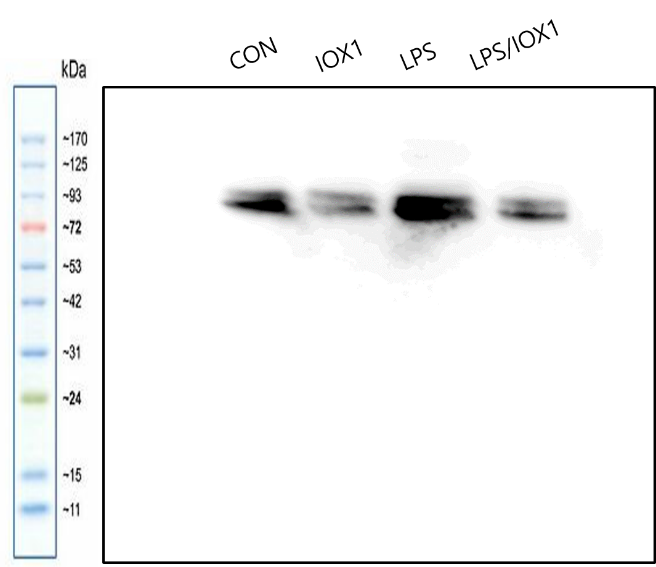

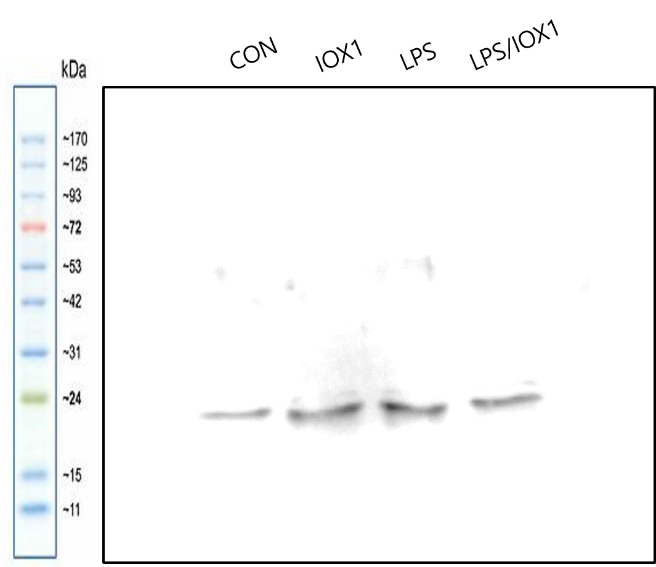


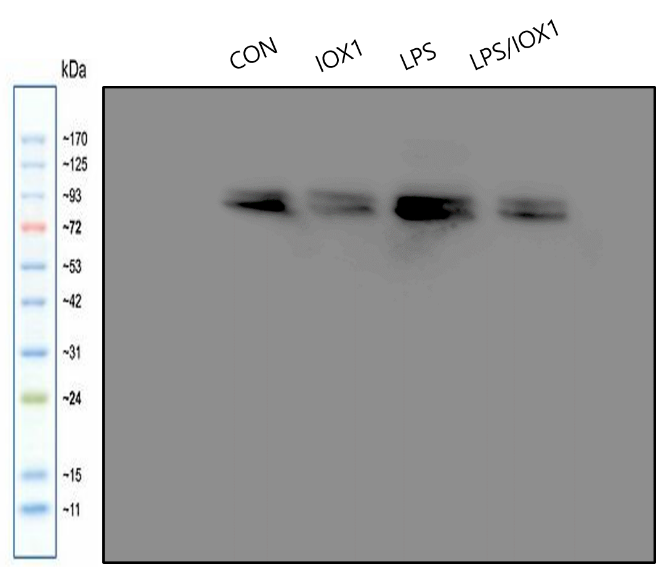

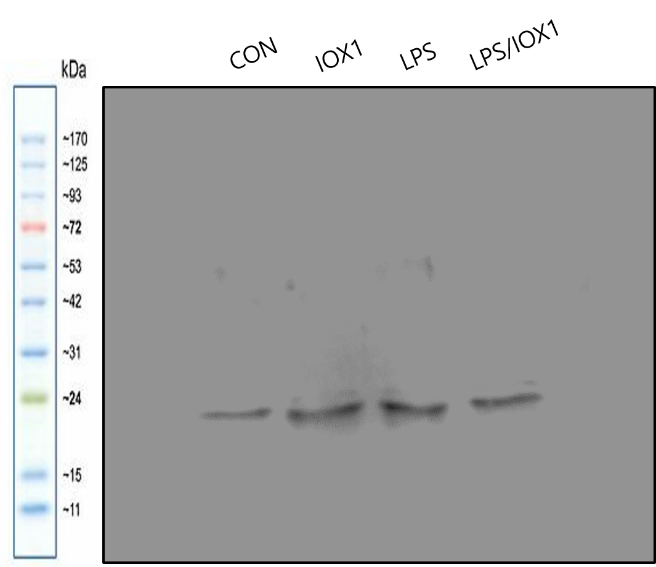


**Fig. S12.** Full-length blots of figure 2C

* In vivo – H3K9me3 * In vivo – H3K36me3


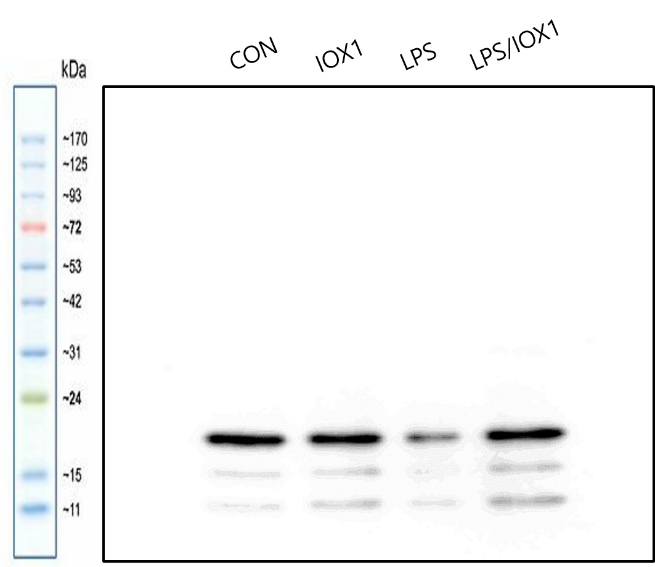

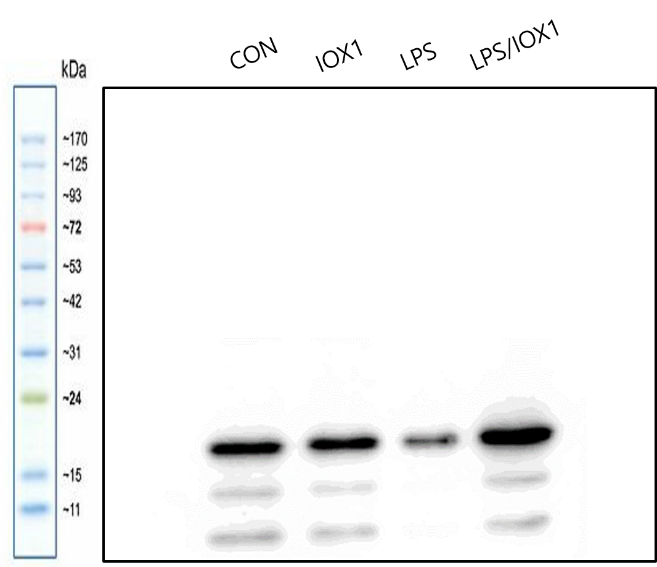


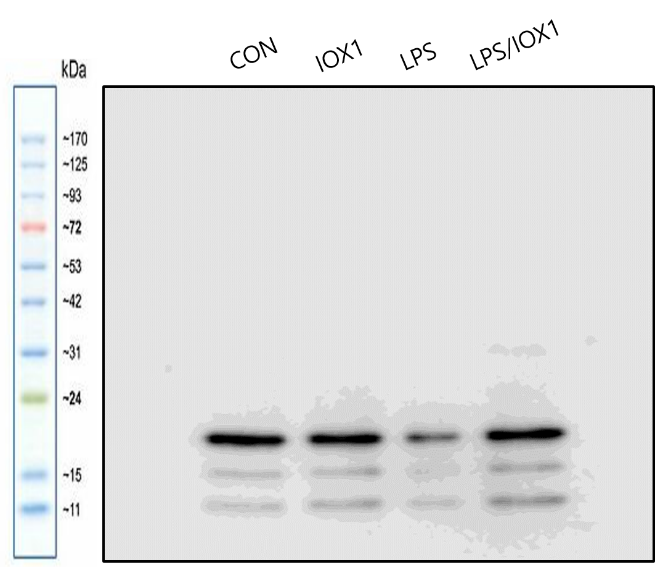

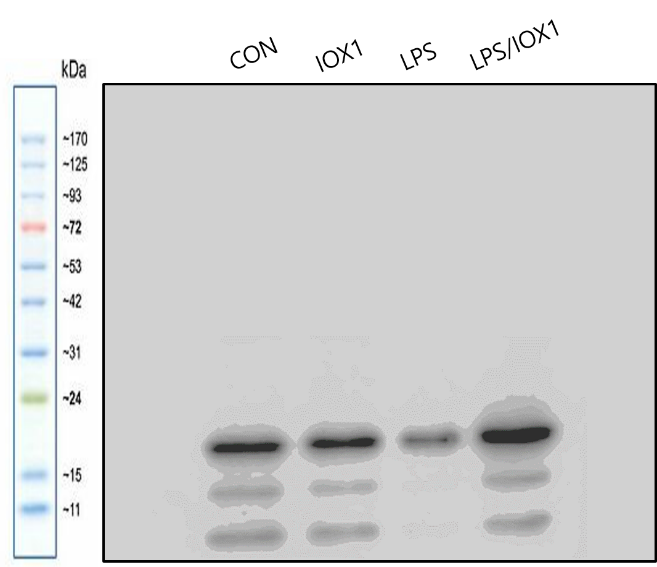


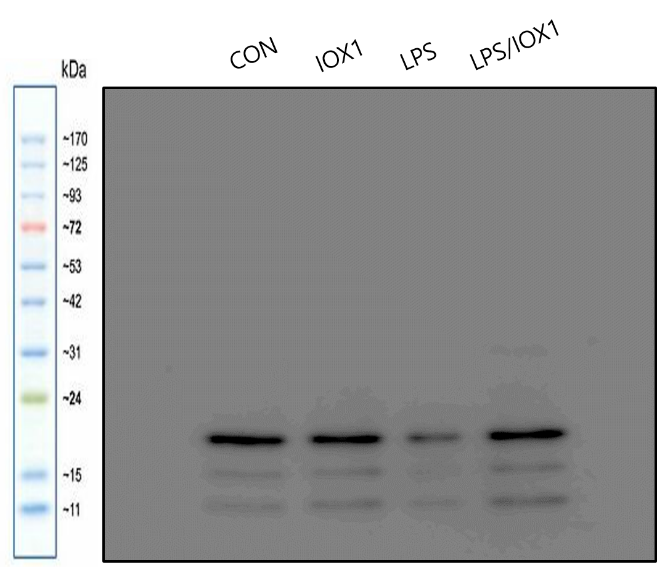

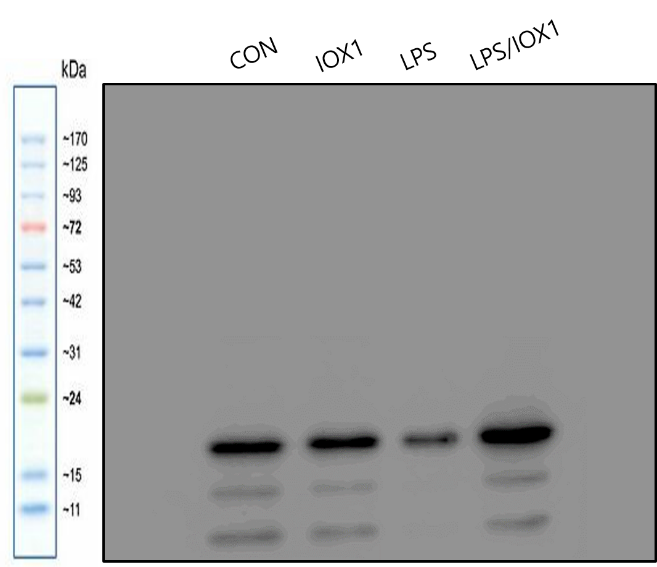


* In vivo – H3


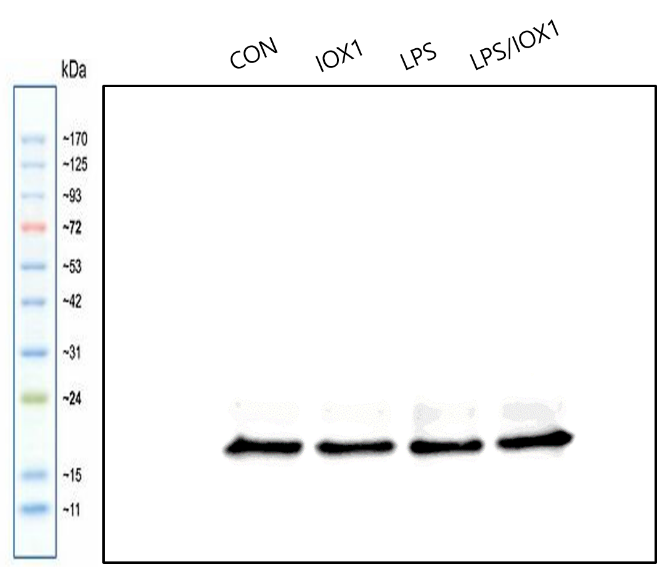


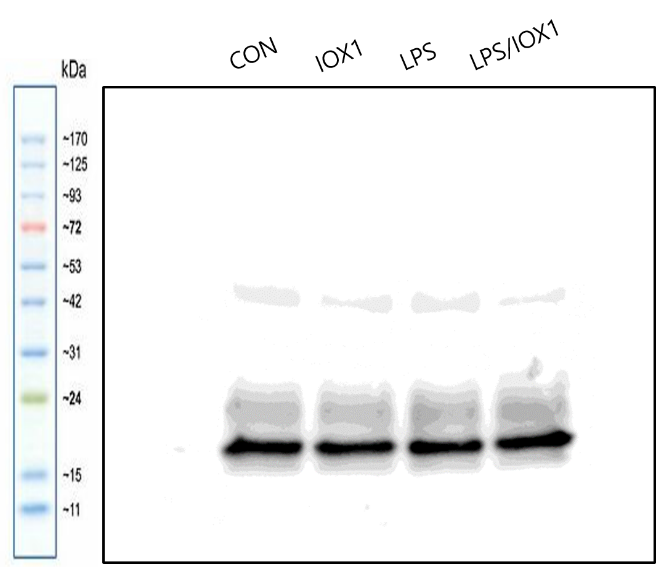


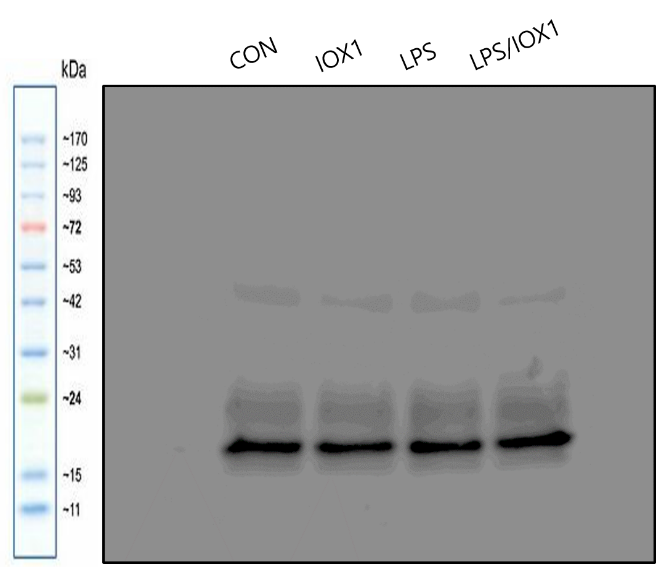


**Fig. S13.** Full-length blots of figure 2D

**
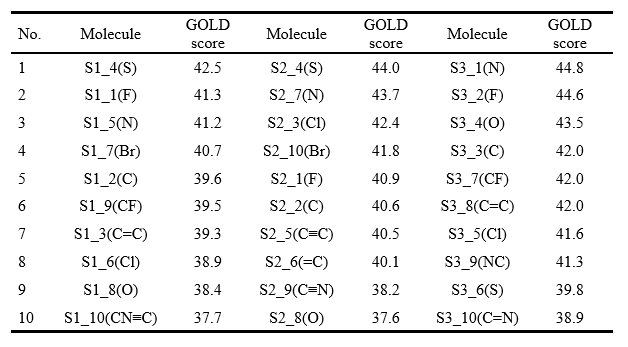
**

**Table S1.** GOLD scores of the different novel derivatives of IOX1.

| Gene name | | 5' -3' primer |
| --- | --- | --- |
| Sequence | | |
| TNF-α | Forward  Reverse | TCGTAGCAAACCACCAAGTG  AGATAGCAAATCGGCTGACG |
| IL-1β | Forward  Reverse | GACCTTCCAGGATGAGGACA  TCCATTGAGGTGGAGAGCTT |
| IL-6 | Forward  Reverse | CTTGGGACTGATGCTGGTGA  TGCAAGTGCATCATCGTTGT |
| IL-12α | Forward  Reverse | CCAGGGTCATTCCAGTCTCT  TCTTCAATGTGCTGGTTTGG |
| IL-10 | Forward  Reverse | GGTTGCCAAGCCTTATCGGA  ACCTGCTCCACTGCCTTGCT |
| b-actin | Forward  Reverse | AAGTGTGACGTTGACATCCG  GATCCACATCTGCTGGAAGG |

**Table S2.** Primers for real-time quantitative PCR
